# Rice Epiphytic Strain *Enterococcus faecalis* YMA3 Improves Corn Stover Silage Quality via Metabolic Regulation Without Shifting the Bacterial Community

**DOI:** 10.3390/ani16162606

**Published:** 2026-08-20

**Authors:** Xuening Luo, Zhifei Zhang, Longxing Hu, Guihua Chen

**Affiliations:** 1 College of Agriculture, Hunan Agricultural University, Changsha 410128, China; a651564994@163.com (X.L.);; 2 Yuelushan Laboratory, Changsha 410128, China

**Keywords:** *Enterococcus faecalis* YMA3, epiphytic lactic acid bacteria, corn stover silage, aerobic stability, feed additive, untargeted metabolomics

## Abstract

Corn stover serves as a key roughage for ruminants, but poor preservation limits its nutritional value. This study evaluates whether *E. faecalis* YMA3, an epiphytic strain from the rice phyllosphere, can improve corn stover silage preservation compared with a commercial *E. faecalis* strain F and an untreated control. We inoculated chopped corn stover with YMA3, strain F, or sterile water, and ensiled the mixtures in vacuum bags for 1, 7, 15, and 45 days. At each time point, we measured fermentation parameters, chemical composition, microbial counts, and aerobic stability and performed untargeted metabolomics on day 45. Both inoculants enhanced preservation relative to the control, as evidenced by higher crude protein content and lactic acid bacteria counts, as well as lower pH, NH_3_-N, and aerobic bacteria counts. Notably, YMA3 outperformed strain F in reducing aerobic bacteria and NH_3_-N levels. Metabolomic analysis identified 309 differential metabolites between YMA3-treated and control silage, including elevated phosphatidylcholine, tauroursodeoxycholic acid, and 3,4-dihydroxyphenylpropionic acid, along with enriched glycerophospholipid, amino acid, and fatty acid metabolism pathways—changes that may underpin improved preservation. In conclusion, *E. faecalis* YMA3 acts as an effective microbial inoculant for corn stover silage, outperforming commercial strain F in protein preservation and aerobic stability by modulating bacterial microbiota and metabolic characteristics. Given the limitation that we tentatively annotated metabolites based on spectral matching rather than authentic standards, follow-up research needs to validate key differential metabolites and evaluate their feeding effects in animal trials. This work provides a preliminary foundation for developing novel microbial feed additives in ruminant production.

## Simple Summary

*E. faecalis* YMA3 improves corn stover silage preservation, extends aerobic stability, and enhances feed safety for ruminant production. Compared with a commercial inoculant, YMA3 delivers modest improvements in protein preservation and aerobic bacterial suppression. Metabolomic profiling reveals differential lipid and organic acid signatures in YMA3-treated silage, including putatively annotated antioxidant candidates—tauroursodeoxycholic acid and 3,4-dihydroxyphenylpropionic acid. Correlation analysis implies their potential roles in inhibiting protein hydrolysis and improving aerobic stability, but further targeted quantification and in vitro functional tests are needed to validate these hypotheses. This study expands the application scope of epiphytic LAB strains as microbial feed additives for ruminant roughage preservation.

## 1. Introduction

Corn stover, the residue remaining after corn grain harvest, is among the most abundant agricultural by-products worldwide, with annual output exceeding 1 billion tons. It acts as an essential roughage for ruminants, especially in areas lacking affordable high-quality forage [1]. Nevertheless, fresh corn stover is unsuitable for direct feeding owing to low digestibility, poor palatability and rapid aerobic spoilage, which impair feed safety and animal productivity [2]. Ensiling with lactic acid bacteria (LAB) inoculants effectively retains nutrients, suppresses spoilage microbes and prolongs storage stability [1,3]. Large quantities of corn stover are still burned or discarded instead of being fed to livestock, mainly because untreated material possesses low digestibility and energy content [3]. Although physical and chemical pretreatment can ameliorate these drawbacks, such approaches entail high costs, potential residue risks and environmental burdens [4]. Microbial ensiling provides an economical and eco-friendly alternative. Under anaerobic conditions, LAB fermentation preserves and improves forage nutrition, conforming to sustainable animal farming by cutting dependence on chemical preservatives and promoting nutrient utilization [1,5].

Commercial LAB inoculants usually adopt allochthonous strains isolated from alien habitats, whose efficiency differs greatly among forage varieties. By comparison, epiphytic LAB naturally residing on plant surfaces adapt well to the substrate, colonize rapidly and facilitate pH decline [6]. Therefore, novel epiphytic strains have attracted extensive attention as candidate microbial additives for specific forages.

Conventional silage analyses, including pH, NH_3_-N and organic acid detection, directly evaluate fermentation quality. Untargeted metabolomics complements these routine indices by unbiased profiling of low-molecular-weight metabolites, enabling identification of metabolic signatures linked to better preservation [7]. This method has been utilized to investigate fermentation processes in alfalfa silage [8], screen biomarkers of aerobic deterioration in whole-crop corn silage [9], and characterize metabolic responses to *Lactobacillus plantarum* inoculation in oat silage [10]. Even so, metabolomic results need verification via targeted quantification and correlation with classical quality indicators to confirm biological functions. Limited research has employed metabolomics to explore epiphytic LAB, particularly when these strains are applied to non-host crops, creating obstacles to nutritional interpretation for ruminant production.

*E. faecalis* YMA3 was isolated from the rice (*Oryza sativa* L.) phyllosphere, and preliminary assays revealed its strong acid-producing capacity and antioxidant activity [11]. Such physiological traits may be functionally relevant to silage preservation. During aerobic exposure after silo opening, oxidative reactions accelerate spoilage and protein degradation. Strains with antioxidant potential could theoretically alleviate oxidative stress, suppress oxidation-driven deterioration and help maintain nutrient stability. Nevertheless, we emphasize that the antioxidant capacity was characterized under pure-culture in vitro conditions. Whether YMA3 exerts comparable antioxidant effects within the complex anaerobic silage matrix, and whether this property contributes to the observed slower pH rise under aerobic conditions, remains to be verified by targeted measurements of oxidation-related metabolites in future trials and it remains unclear whether these advantageous characteristics can be maintained when applied to corn stover, a C4 grass that differs substantially from C3 rice in cell wall structure and carbohydrate profile [12]. The identity of plant hosts can shape the colonization of epiphytic lactic acid bacteria by altering surface chemical properties, exudate components and indigenous microbial communities [13]. Despite these botanical discrepancies, preliminary physiological evaluation indicated that *E. faecalis* YMA3 possesses promising traits for silage inoculation, including robust anaerobic adaptability, efficient lactic acid biosynthesis, acid tolerance, and broad-spectrum utilization of soluble carbohydrates, and the strain does not display strict host specificity. Accordingly, we hypothesized that YMA3 could rapidly proliferate in chopped corn stover under anaerobic ensiling conditions, establish dominance within the bacterial community during the early fermentation stage, accelerate pH reduction via lactic acid accumulation, suppress the proliferation of detrimental microorganisms, and ultimately improve fermentation quality and nutrient preservation of corn stover silage. Testing YMA3 on this non-host substrate is therefore critical to clarify whether its beneficial functions are host-dependent or widely applicable across grass species, which would greatly broaden its potential as a microbial feed additive for diverse ruminant production systems.

Existing studies rarely assess the efficacy of rice-derived epiphytic LAB on corn stover, and few adopt untargeted metabolomics to decipher the molecular mechanisms responsible for improved silage preservation.

Accordingly, we evaluated the potential of YMA3 as a microbial feed additive in corn stover silage, with an untreated control and a commercial *E. faecalis* strain (Strain F) as references. The silage was ensiled for 1, 7, 15, and 45 days, and fermentation quality, chemical composition, microbial counts, and aerobic stability were determined at each time point. Additionally, Untargeted metabolomic analysis was performed on samples from the control group (CK) and the YMA3-inoculated group on day 45 of ensiling to characterize metabolic pathways modulated by YMA3 inoculation. We hypothesized that (1) YMA3 inoculation would significantly improve fermentation parameters (lower pH, higher lactic acid bacteria counts, and reduced NH_3_-N) compared to the untreated control; (2) YMA3 would demonstrate superior protein preservation and aerobic stability relative to both the control and commercial strain F; (3) YMA3 treatment would alter the metabolite profile of corn stover silage, with significant enrichment in lipid and amino acid metabolism pathways, offering preliminary insights into its preservation mechanisms.

## 2. Materials and Methods

To comprehensively clarify how *E. faecalis* YMA3 regulates corn stover fermentation and feeding value for ruminants, multiple chemical, fermentation and microbial indicators were selected with clear nutritional and preservation-oriented purposes.

Dry matter (DM) loss was determined to quantify total nutrient retention during ensiling; DM loss directly reflects whether energy substances are degraded and lost, and excessive DM loss reduces the total digestible energy intake of ruminants. Crude protein was measured because crude protein is the primary limiting nutrient for roughage-fed ruminants; protein degradation driven by spoilage bacteria produces NH_3_-N, lowering rumen microbial protein synthesis efficiency and animal production performance. Water-soluble carbohydrate (WSC) is the core fermentation substrate of lactic acid bacteria. Detecting dynamic changes in WSC can explain the acidification rate difference between inoculated groups and the untreated control, and further interpret how high moisture triggers acetic acid and butyric acid accumulation in early natural fermentation. Neutral detergent fiber (NDF), acid detergent fiber (ADF), hemicellulose and lignin were evaluated to judge fiber digestibility for ruminants; fiber composition determines roughage physical fill effect and rumen degradation rate. Fermentation end-products (organic acids, pH, NH_3_-N) were used to assess the inhibition of harmful microorganisms. Finally, metabolomics analysis was adopted to reveal the unique metabolic regulation of YMA3, rather than only focusing on conventional lactic acid fermentation, which helps explain potential advantages in antioxidant capacity and long-term storage stability for ruminant feeding. All indicators were screened centering on two core goals: maximizing corn stover nutrient preservation during ensiling and optimizing its rumen nutritional availability for ruminants.

### 2.1. Silage Making

We used waxy corn (*Zea mays* L. var. ceratina cv. ‘Xiangnuo White Pear’) as the experimental material. After threshing the corn and chopping the stover to approximately 2 cm in length, corn stover consisting of stalks and leaves, we packed the stover into polyethylene silage bags (500 g per bag). The experiment included three treatment groups: (a) Control group (sterile deionized water, 10 mL/kg FM), (b) *E. faecalis* strain YMA3 group (1 × 10^6^ CFU/g FM in 10 mL sterile deionized water), and (c) commercial *E. faecalis* strain F group (1 × 10^6^ CFU/g FM in 10 mL sterile deionized water). We selected the inoculation rate of 1 × 10^6^ CFU/g FM based on commercial recommendations for LAB silage inoculants (Pioneer^®^ 11C33, Corteva Agriscience, Indianapolis, IN, USA; recommended rate: 1 × 10^5^–1 × 10^6^ CFU/g FM). Prior to inoculation, we cultured YMA3 and strain F in de Man, Rogosa, and Sharpe (MRS) broth at 37 °C for 18 h, harvested the cells by centrifugation (8000× *g*, 10 min, 4 °C), and washed them twice with sterile deionized water to remove residual culture medium. We sprayed the control group with an equal volume of sterile deionized water. Independent silo bags were prepared for each sampling time point, and each bag was opened only once for sampling to avoid repeated disturbance to the anaerobic environment. One silage bag represented one experimental unit. Total number of silage bags was 72. We collected samples in triplicate from each treatment after storing them at ambient temperature (25 ± 2 °C) for 1, 7, 15, and 45 days. Subsequently, we determined microbial counts, fermentation quality, nutritional composition, and aerobic stability of the samples.

### 2.2. Measurements and Analytical Methods

#### 2.2.1. Microbial Counting

We counted lactic acid bacteria (LAB), aerobic bacteria, yeast, and mold in both the raw material and silage with reference to [14] and made appropriate modifications. Ten grams of silage sample were blended with 90 mL of sterile 0.85% NaCl solution in a sterile homogenization bag and homogenized for 2 min using a stomacher. Serial 10-fold dilutions were prepared with sterile saline. Aliquots (0.1 mL) of appropriate dilutions were spread onto corresponding selective agar plates and incubated under specified conditions. Lactic acid bacteria (LAB) were cultured on de Man, Rogosa and Sharpe (MRS) agar at 37 °C for 48 h under anaerobic conditions. Aerobic bacteria were incubated on nutrient agar at 28 °C for 48 h under aerobic conditions. Mold and yeast were grown on potato dextrose agar (PDA) at 28 °C for 72 h. Colonies within the countable range (30–300 CFU per plate) were counted. The results were expressed as colony-forming units per gram of fresh silage (CFU/g FM) and transformed to log_10_ CFU/g FM for statistical analysis.

#### 2.2.2. Nutritional Indices

A 200 g sample of silage was subjected to enzyme inactivation and oven-dried at 65 °C to a constant weight for DM determination. The dried sample was ground to pass through a 1 mm sieve and subsequently through a 0.425 mm sieve for chemical analyses. Contents of crude protein (Kjeldahl method), water-soluble carbohydrates (Anthrone method), neutral detergent fiber (NDF, ANKOM200 method with α-amylase and sodium sulfite), acid detergent fiber (ADF, ANKOM200 method), and acid detergent lignin (ADL, 72% sulfuric acid method) were determined in triplicate for each parameter [15].

Cellulose content (% DM) = ADF (% DM) − residual content after 72% sulfuric acid treatment

Hemicellulose content (% DM) = NDF (% DM) − ADF (% DM)

#### 2.2.3. Fermentation Indices

20 g fresh silage subsample was combined with 180 mL distilled water (1:9 ratio) and soaked at 4 °C for 24 h. The mixture was filtered through four layers of cheesecloth to acquire silage aqueous extracts. The pH of each filtrate was measured at ambient temperature using a calibrated digital pH meter [15]. Concentrations of lactic acid, acetic acid, propionic acid and butyric acid were quantified by high-performance liquid chromatography (HPLC). To ensure the reproducibility of untargeted metabolomics analysis, the detailed LC-MS/MS chromatographic parameters are summarized in Table 1.

#### 2.2.4. pH Dynamics Under Aerobic Exposure

The remaining silage after DM sampling was loosely packed into sterile plastic buckets, covered with two layers of sterile gauze to allow air penetration while preventing contamination, and stored at ambient temperature (25 ± 2 °C). At 12, 48, 96, and 144 h of aerobic exposure, 20 g of silage was mixed with 180 mL of distilled water and extracted at 4 °C for 24 h (in triplicate). The pH of the extract was then determined using a pH meter (Mettler Toledo FE28, Columbus, OH, USA) [16].

#### 2.2.5. Metabolite Profiling Analysis

Immediately upon opening, samples were transferred to centrifuge tubes, flash-frozen in liquid nitrogen, and stored at −80 °C. Metabolomic samples comprised silage fermented for 45 days, including a control group and a YMA3-treated group (YMA3), with six biological replicates per group. Untargeted metabolomics analysis was performed by Shanghai Personal Biotechnology Co., Ltd. (Shanghai, China) to screen metabolite variation linked to silage preservation. It should be clearly stated that untargeted metabolomics only provides relative abundance based on spectral matching against public databases rather than absolute quantitative data. All differential metabolites and enriched pathways obtained herein are statistical associations instead of confirmed biological mechanisms; authentic standard-based LC-MS/MS quantification and in vitro activity tests are necessary to verify the functions of candidate metabolites.

#### 2.2.6. Statistical Analysis

Experimental data were compiled using Microsoft Excel. Analysis of variance (ANOVA) was performed using SPSS Statistics 26.0, followed by Duncan’s multiple range test for multiple comparisons. Graphs were generated using Origin 2024. Statistical significance was set at *p* < 0.05. Results are presented as the mean ± standard deviation (SD). For metabolomic data, principal component analysis (PCA) was performed to assess group separation, and differential metabolites were identified based on variable importance in projection (VIP) scores > 1 and *p* < 0.05. Pearson correlation coefficients were calculated between metabolite relative abundances and fermentation parameters to explore potential associations, with significance determined at *p* < 0.05. KEGG enrichment analysis was conducted to identify potentially affected metabolic pathways, with the caveat that pathway assignments based on untargeted data are predictive rather than confirmatory. To further interpret the relationship between bacterial taxa and differential metabolites, Pearson correlation coefficients between the relative abundance of dominant genera and the top 50 significantly altered metabolites were calculated, and a correlation network was visualized using the igraph package in R. PICRUSt2 was adopted to predict potential functional pathways of bacterial communities based on 16S rRNA sequencing data. PICRUSt2 analysis was performed to predict the functional potential of microbial communities in corn stover silage. Based on 16S rRNA gene sequencing data, it infers metabolic functions of uncultured microorganisms via phylogenetic comparison with reference genomes. The main output metrics include predicted KEGG orthologs (KOs), pathway abundances, and enzyme commission (EC) numbers, which help elucidate functional changes in microbial communities in YMA3-treated silage and link microbial structure to metabolic characteristics and preservation performance.

## 3. Results

### 3.1. Chemical Composition and Composition of Epiphytic Microorganisms of Raw Materials

The corn stover dry matter (DM) content was 22.46 ± 0.41%, containing 13.59 ± 0.37% crude protein, 11.27 ± 0.36% water-soluble carbohydrates (WSC), 53.33 ± 0.28% neutral detergent fiber (NDF), 26.69 ± 0.29% acid detergent fiber (ADF), 2.00 ± 0.26% acid detergent lignin (ADL), 26.64 ± 0.18% hemicellulose (HC), 24.69 ± 0.41% cellulose (CL), and 8.50 ± 0.13% ash, all expressed as percentages of dry matter. Lactic acid bacteria (LAB), aerobic bacteria, yeast, and mold were 3.38 ± 0.28 log10 CFU/g FM, 5.23 ± 0.55 log10 CFU/g FM, and 3.92 ± 0.22 log10 CFU/g FM, 4.54 ± 0.31 log10 CFU/g FM, respectively. All the above indicators were determined with three biological replicates.

### 3.2. The Effects of LAB on the Nutritional Quality of Corn Stover Silage

Table 2 illustrates the effects of LAB inoculation on the nutritional quality of corn stover silage. No significant difference in dry matter (DM) content was observed among treatments at any time point (*p* > 0.05). With prolonged ensiling time, DM contents in the Control and F groups showed a decreasing trend, with significant reductions observed between day 7 and day 15 (Control: 21.20% to 20.43% DM, *p* < 0.05; F: 21.83% to 20.92% DM, *p* < 0.05), whereas the YMA3 group maintained stable DM content throughout the ensiling period (day 7: 21.41% DM; day 15: 20.58% DM; *p* > 0.05). Similarly, crude protein content decreased significantly with prolonged ensiling in the Control group (day 1: 13.41% DM; day 45: 11.62% DM; *p* < 0.05) and the F group (day 1: 13.34% DM; day 45: 12.14% DM; *p* < 0.05 for day 1 vs. day 45), whereas the YMA3 group maintained stable crude protein levels throughout the ensiling period (day 1: 13.20% DM; day 45: 12.35% DM; *p* > 0.05). On day 45, crude protein content in the Control group (11.62 ± 0.04% DM) was significantly lower than in the YMA3 (12.35 ± 0.36% DM) and F (12.14 ± 0.15% DM) groups (*p* < 0.05), with no significant difference between YMA3 and F (*p* > 0.05).

Water-soluble carbohydrate (WSC) content decreased significantly with ensiling time across all treatments (*p* < 0.05). Notably, WSC content in the control group was significantly higher than that in the inoculated groups at all time points (*p* < 0.05), indicating more efficient substrate utilization by LAB in the inoculated treatments. Notably, although NDF content decreased significantly over the ensiling period (*p* < 0.05), the F group exhibited significantly higher NDF content (53.55 ± 0.58% DM) than the Control group (51.04 ± 1.17% DM) on day 1 (*p* < 0.05). However, no significant differences in NDF content were detected among groups on days 7, 15, and 45 (*p* > 0.05). On day 45, no significant difference in acid detergent fiber (ADF) content was observed between the Control (27.74 ± 0.57% DM) and YMA3 (28.47 ± 0.08% DM) groups (*p* > 0.05), suggesting that exogenous LAB inoculation did not significantly reduce fiber content. At each sampling time point, no significant differences were observed in acid detergent lignin (ADL), hemicellulose, or ash contents among groups (*p* > 0.05).

### 3.3. The Effects of LAB on the Fermentation Quality of Corn Stover Silage

Table 3 illustrates the effects of lactic acid bacteria inoculation on the fermentation quality of corn stover silage. In the present study, the pH of the inoculated groups was significantly lower than in the control group at 45 days of ensiling (YMA3: 3.83 ± 0.00; F: 3.82 ± 0.00; Control: 3.90 ± 0.01; *p* < 0.05). Inoculation with *E. faecalis* YMA3 significantly decreased NH_3_-N content compared to both the control and strain F. On day 45, the NH_3_-N content in the YMA3 group (1.99 ± 0.04 g/kg DM) was significantly lower than that in group F (2.47 ± 0.06 g/kg DM; *p* < 0.05) and the control group (2.64 ± 0.04 g/kg DM; *p* < 0.05), effectively inhibiting the degradation of crude protein in silage. At 45 days of ensiling, the YMA3 group exhibited significantly higher LAB populations (6.58 ± 0.17 log10 CFU/g FM) and lower aerobic bacterial counts (4.87 ± 0.19 log10 CFU/g FM) than the F group (LAB: 6.43 ± 0.13 log10 CFU/g FM; aerobic bacteria: 5.47 ± 0.07 log10 CFU/g FM) and the Control group (LAB: 5.72 ± 0.16 log10 CFU/g FM; aerobic bacteria: 5.90 ± 0.07 log10 CFU/g FM) (*p* < 0.05). At 15 days of ensiling, the lactic acid content in the Control group (19.78 ± 0.27% DM) was significantly higher than in the F group (15.17± 2.80% DM; *p* < 0.05), likely due to continued activity of epiphytic LAB in the absence of rapid pH decline. At 45 days, no significant difference in lactic acid content was observed between the YMA3 (13.37 ± 1.74% DM) and F (17.78 ± 1.66% DM) groups (*p* > 0.05). Acetic acid was detected only in the Control group at 15 days (1.46 ± 0.05% DM), suggesting heterolactic fermentation activity by epiphytic microbiota before the establishment of dominant homolactic fermentation. No significant difference in propionic acid content was observed among treatments at any time point (*p* > 0.05). On day 1, butyric acid content in the Control group (0.51 ± 0.14% DM) was significantly higher than in the F group (0.15 ± 0.11% DM; *p* < 0.05); however, no significant differences were detected at other time points (*p* > 0.05).

It is noteworthy that while YMA3 outperformed strain F in specific parameters (aerobic bacterial counts: 10.97% lower; NH_3_-N: 19.43% lower), both inoculants achieved similar pH reduction (YMA3: 3.83; F: 3.82) and lactic acid production (YMA3: 13.37% FM; F: 17.78% FM; *p* > 0.05).

### 3.4. The Effects of LAB on the Aerobic Stability of Corn Stover Silage

**Figure 1:**
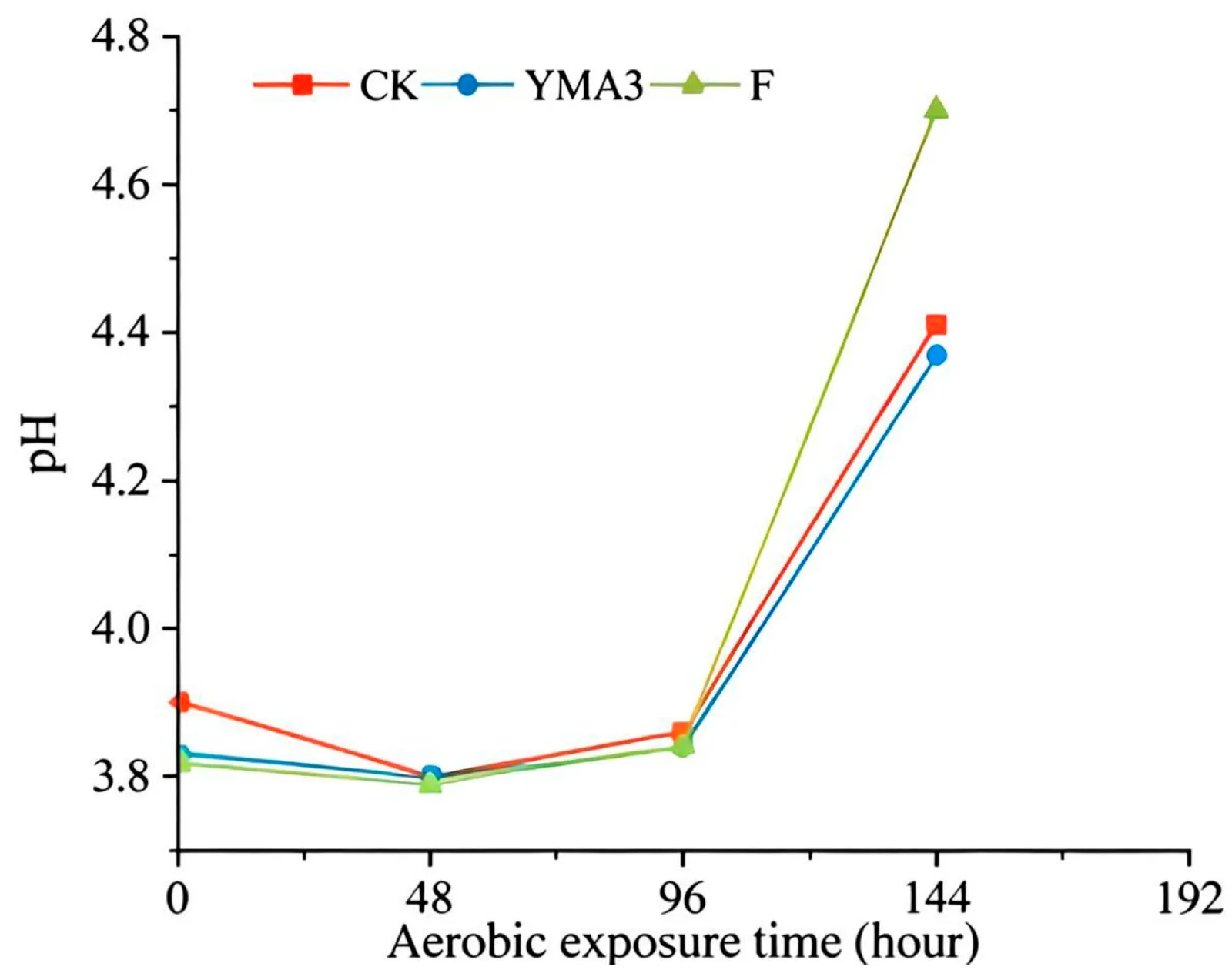
Changes in pH of corn stover silage during aerobic exposure (0–144 h) following 45 days of ensiling, indicating feed safety and spoilage risk for ruminant production. The red, blue, and green lines represent the Control, YMA3, and F groups, respectively. Data points indicate the means of three biological replicates; CK = Control.

Aerobic deterioration of silage has long been a major concern for livestock producers, as it not only impairs nutritional value but also poses risks to animal and human health [2,17]. This study found that the pH of corn stover silage increased significantly during aerobic exposure, with pH values in the YMA3 and F groups increasing by 14.10% (from 3.83 to 4.37) and 23.04% (from 3.82 to 4.70), respectively, after 144 h of aerobic exposure (Figure 1).

### 3.5. The Effects of Different Treatments and Ensiling Durations on Both Fermentation and Nutritional Parameters of Corn Stalks

As shown in Table 4, the interactions between pH value, acetic acid content, ammonia nitrogen content, lactic acid bacteria count, aerobic bacteria count, ensiling duration, and microbial inoculant were highly significant (*p* <0.01); the interaction between soluble carbohydrates and ensiling duration and microbial inoculant was significant (*p* <0.05). For all other indicators, the interactions with ensiling duration and microbial inoculant were not significant (*p*> 0.05).

### 3.6. The Effects of Inoculating E. faecalis YMA3 on the Microbial Composition of Corn Stover Silage

**Figure 2:**
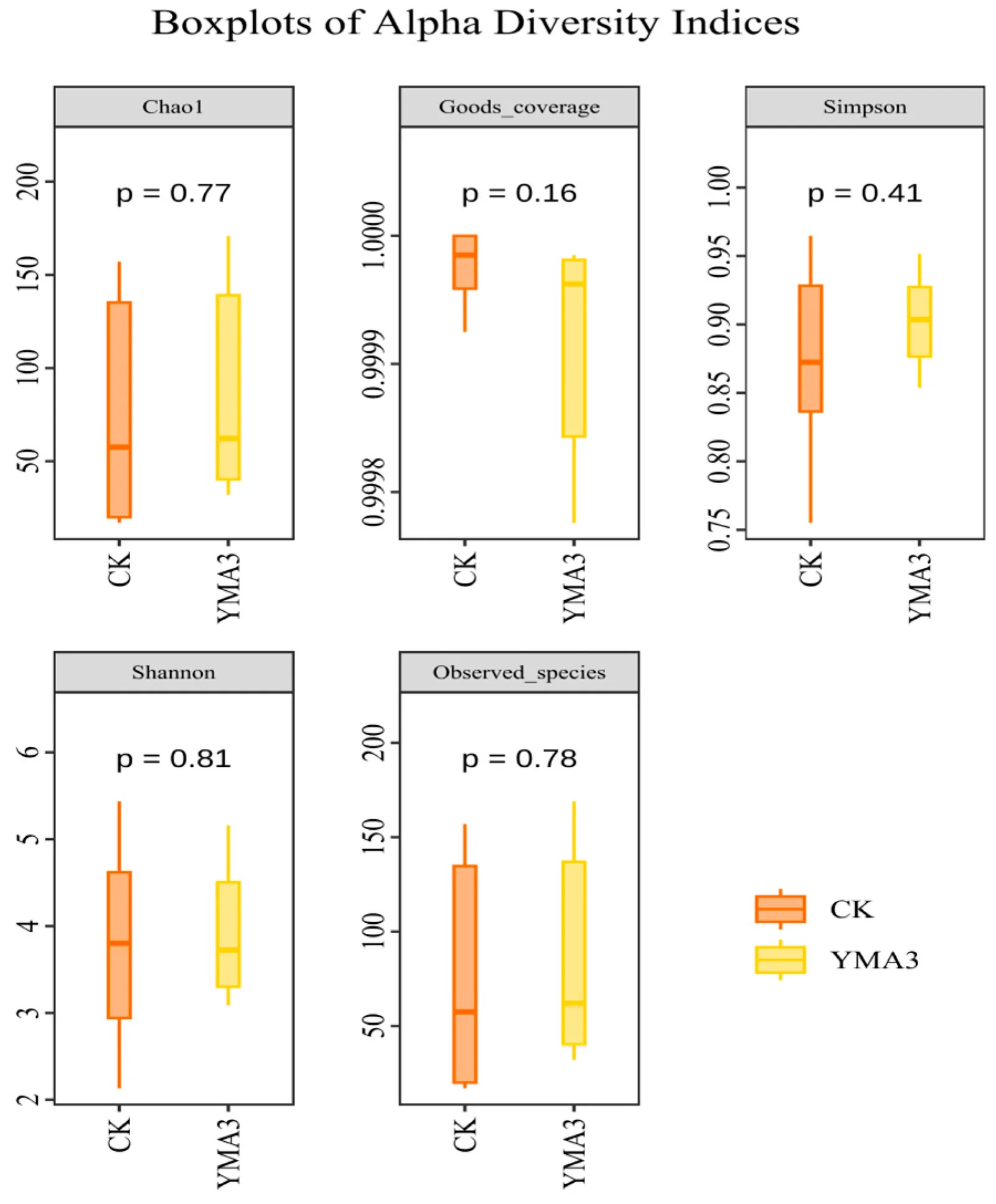
Effects of *E. faecalis* YMA3 inoculation on the bacterial community of corn stover silage. Alpha diversity indices of bacterial communities; CK = Control.

**Figure 3:**
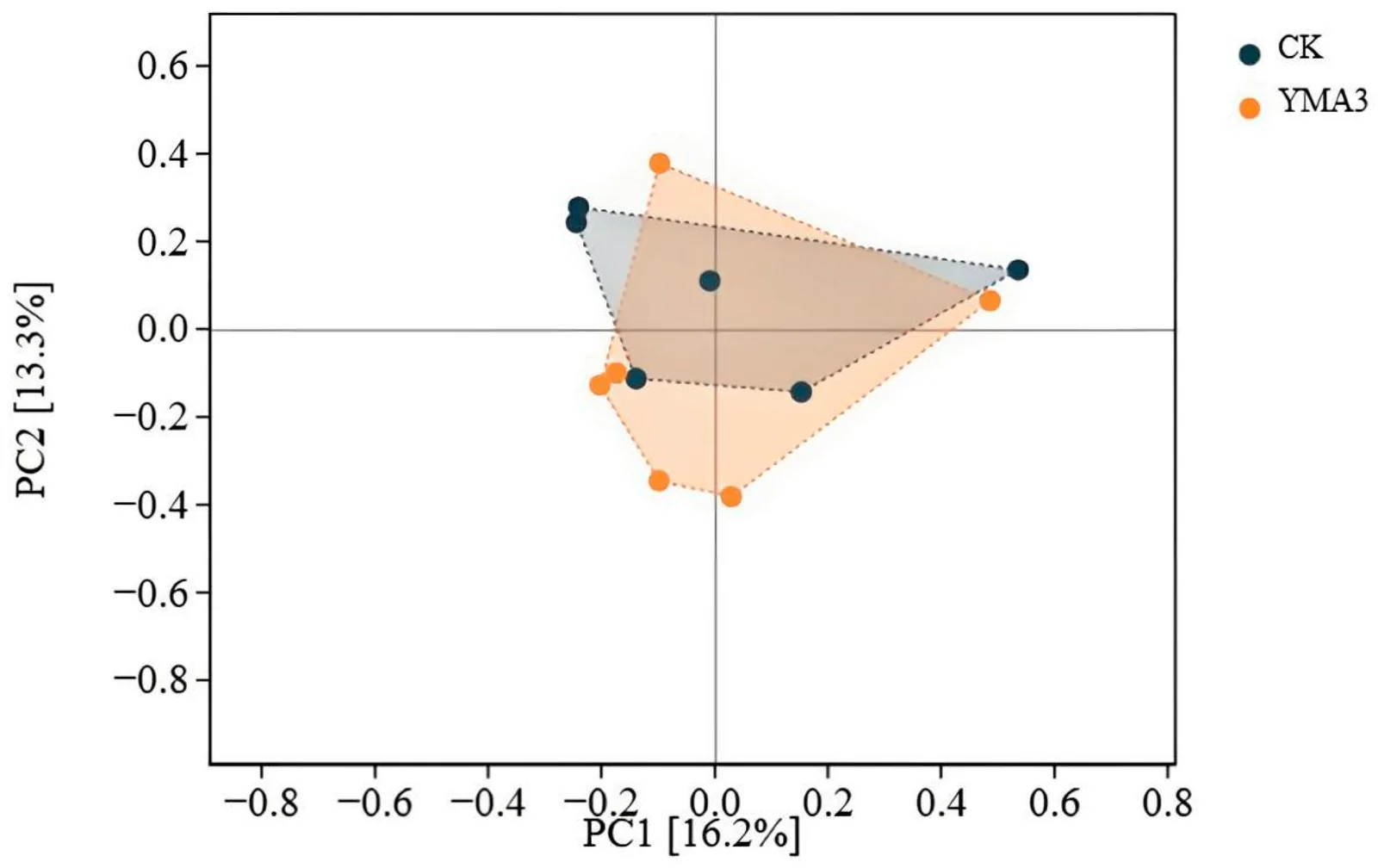
Principal coordinate analysis (PCoA) based on Bray–Curtis distance, with PC1 and PC2 explaining 16.2% and 13.3% of the total variation, respectively. Adonis analysis revealed no significant difference between the control and YMA3 groups (*p* = 0.489). Abbreviations: CK, non-inoculated control group; YMA3, *E. faecalis* YMA3-inoculated group; CK = Control.

**Figure 4:**
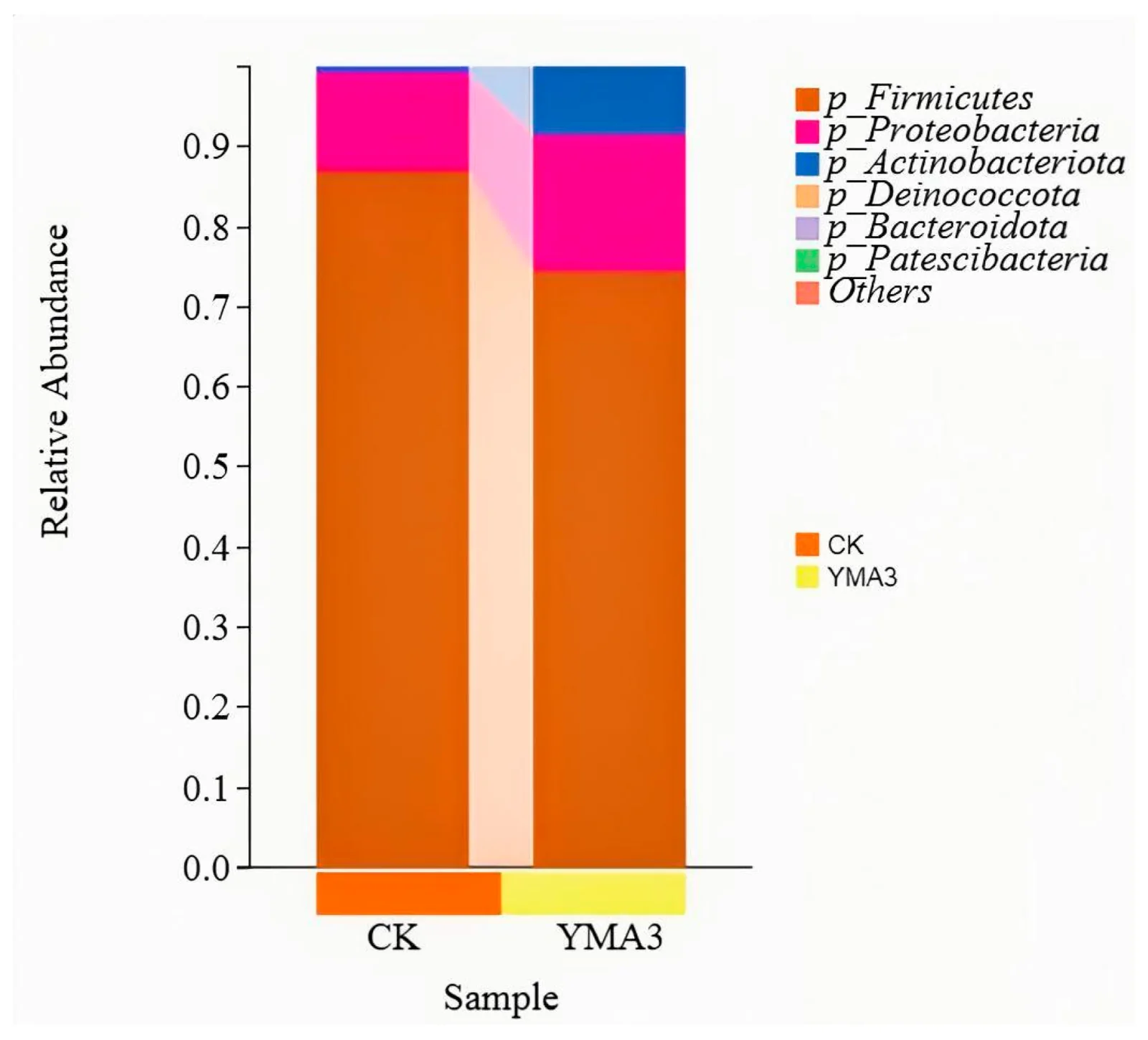
Relative abundances of bacterial phyla in corn stover silage with and without *E. faecalis* YMA3 inoculation. The *X*-axis indicates treatment groups, while the *Y*-axis shows the corresponding relative abundance (%); CK = Control.

Inoculating with YMA3 had no significant effects on the Chao1, Shannon, and Simpson indices of the bacterial community in corn stover silage (*p* > 0.05), indicating no significant differences in bacterial community richness and diversity among treatments (Figure 2). PCoA was performed based on the Bray–Curtis distance matrix. The first principal coordinate (PC1) explained 16.2% of the total variation, and the second principal coordinate (PC2) explained 13.3% of the variation. Adonis analysis revealed no significant difference between the control group and the YMA3 group (*p* = 0.489, R^2^ = 0.0904) (Figure 3). Firmicutes was found to be the most abundant phylum in ensiled materials (Figure 4).

### 3.7. The Effects of Inoculating E. faecalis YMA3 on the Metabolite Composition of Corn Stover Silage

**Figure 5:**
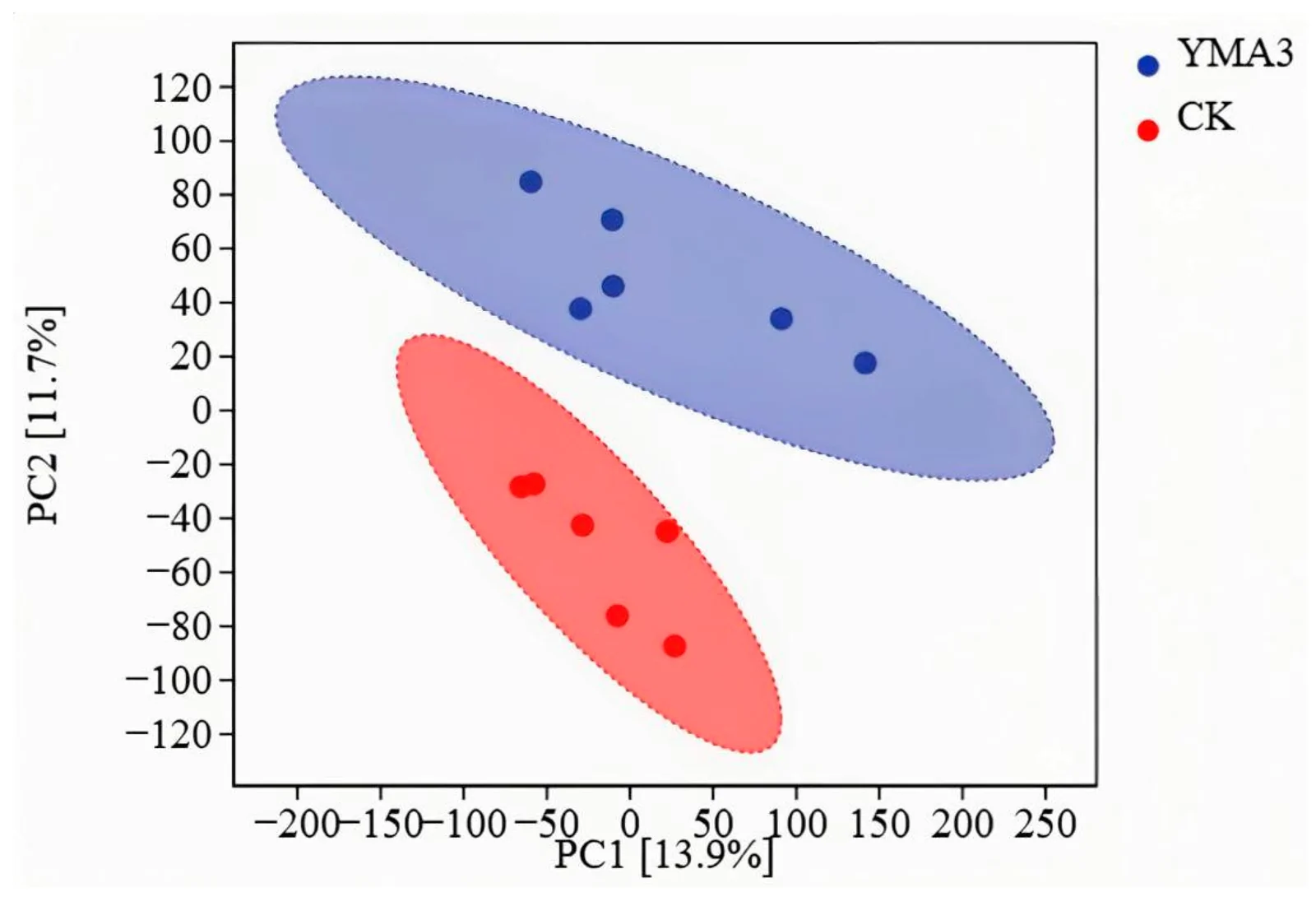
Principal component analysis (PCA) of metabolite profiles in corn stover silage inoculated with *E. faecalis* YMA3. The blue and red dashed circles represent the 95% confidence regions for the CK (control) and YMA3 groups, respectively; CK = Control.

**Figure 6:**
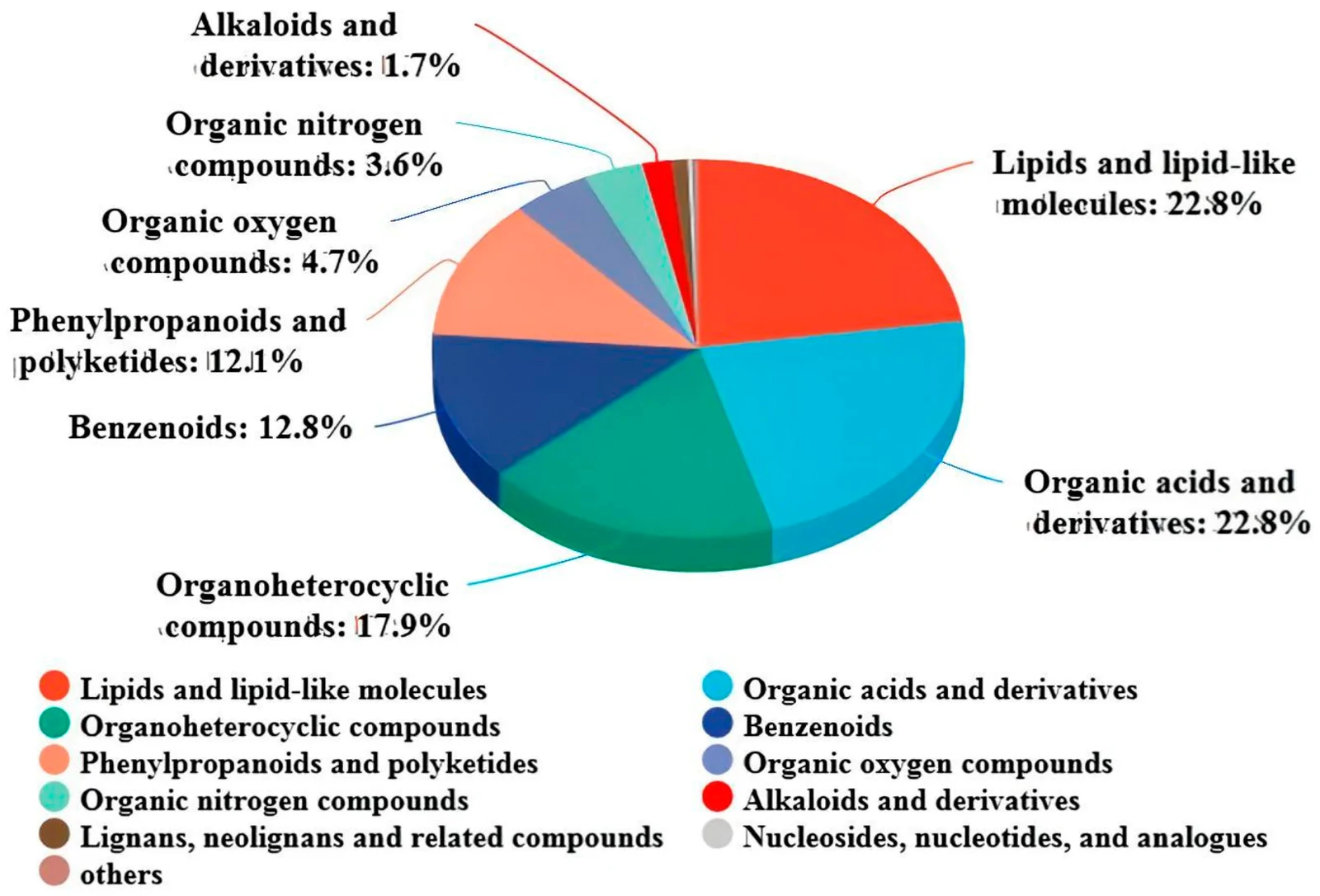
Relative abundances of metabolite categories in corn stover silage with and without inoculation with *E. faecalis* YMA3.

Principal component analysis (PCA) of metabolites revealed a clear separation in metabolic profiles between the Control and YMA3 groups (PC1: 16.2%; PC2: 13.3%), indicating that YMA3 is the primary driver modulating the metabolome of corn stover silage. This finding further suggests that YMA3 inoculation induces substantial metabolic alterations during ensiling, warranting subsequent detailed metabolomic analysis (Figure 5). Classification of detected metabolites revealed that lipids and lipid-like molecules, as well as organic acids and their derivatives, represented the largest proportions, each accounting for 22.8% of all detected metabolites. The former category included fatty acid-related compounds such as tauroursodeoxycholic acid, anomanolide D, and 1-heptadecanoyl-sn-glycero-3-phosphocholine, while the latter comprised organic acids including 3-acetamido-3-(4-fluorophenyl) propanoic acid and Tyr-Phe (tyrosine–phenylalanine). Organic heterocyclic compounds represented the third largest category (17.9%), including 6-bromoindole-3-carboxaldehyde, 6-bromo-4-chloro-8-fluoroquinoline, and related compounds. Additionally, phenylpropanoids and polyketides accounted for 12.8% and 12.1% of identified metabolites, respectively (Figure 6).

**Figure 7:**
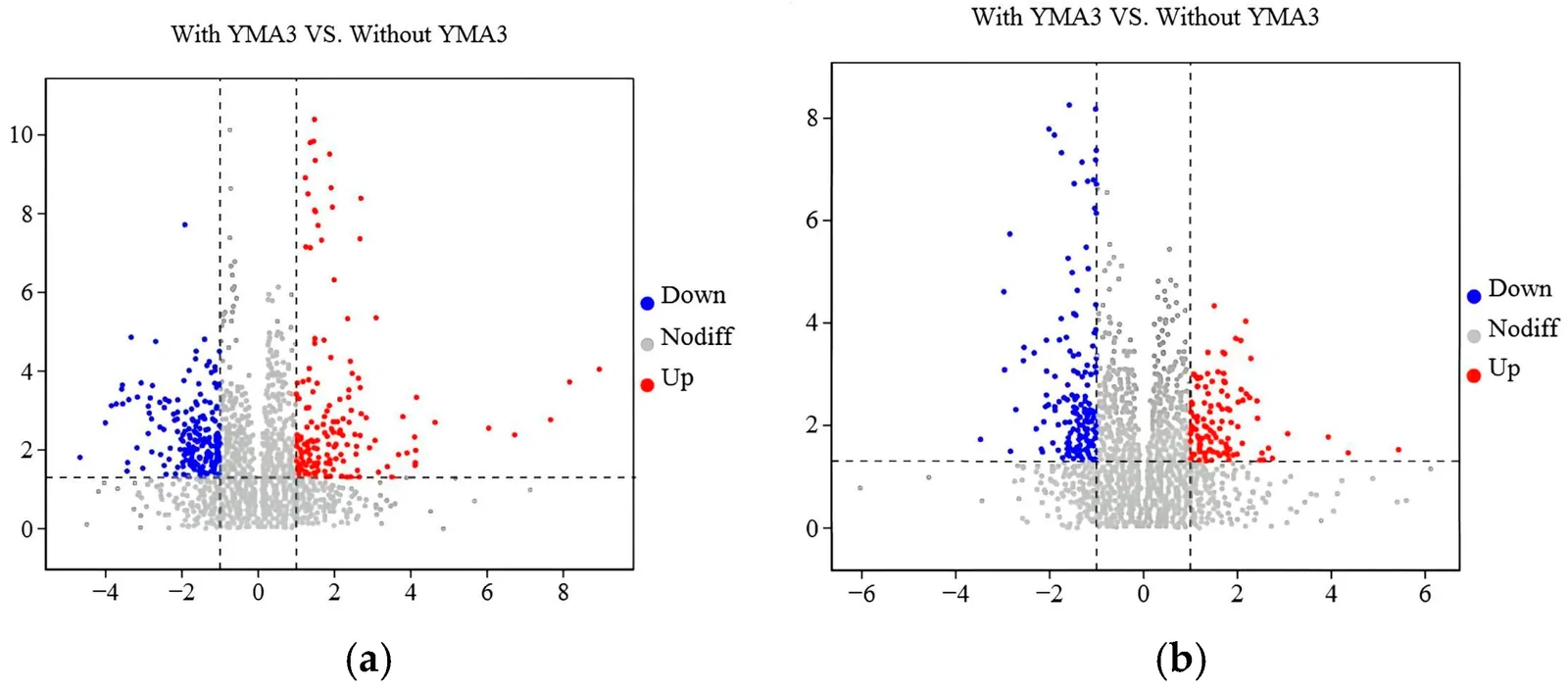
Volcano plot showing differentially abundant metabolites between the non-inoculated control group and the *E. faecalis* YMA3-inoculated group of corn stover silage. Dashed lines indicate the thresholds of fold change (FC > 2 or < 0.5) and *p*-value (*p* < 0.05). (**a**) Positive ion mode. (**b**) Negative ion mode.

**Figure 8:**
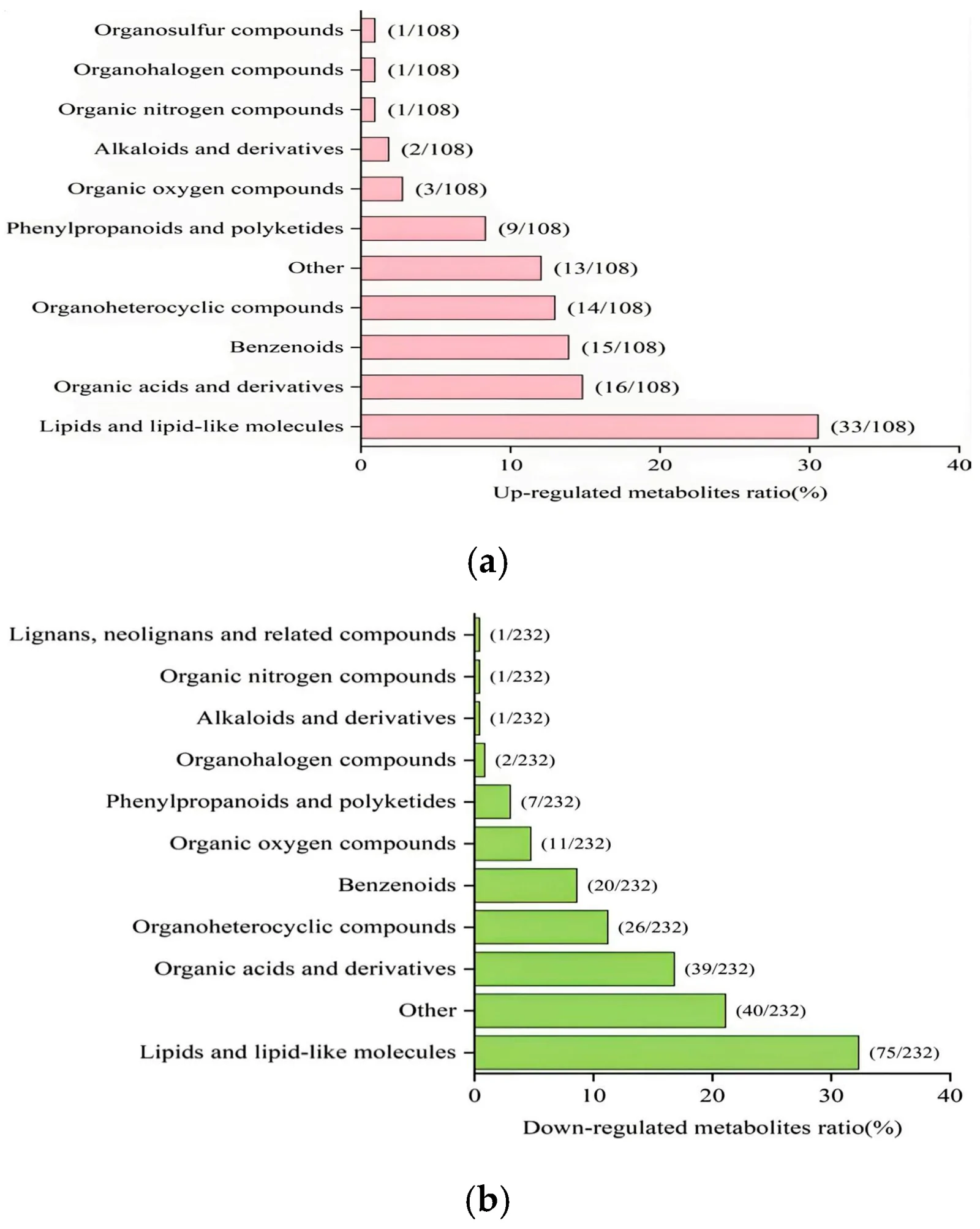
Proportions of metabolite categories among (**a**) up-regulated and (**b**) down-regulated metabolites in YMA3-inoculated corn stover silage. The *X*-axis indicates the percentage proportion of metabolites in each category. Values in parentheses denote the ratio of metabolite counts in each category to the total number of differentially abundant metabolites.

**Figure 9:**
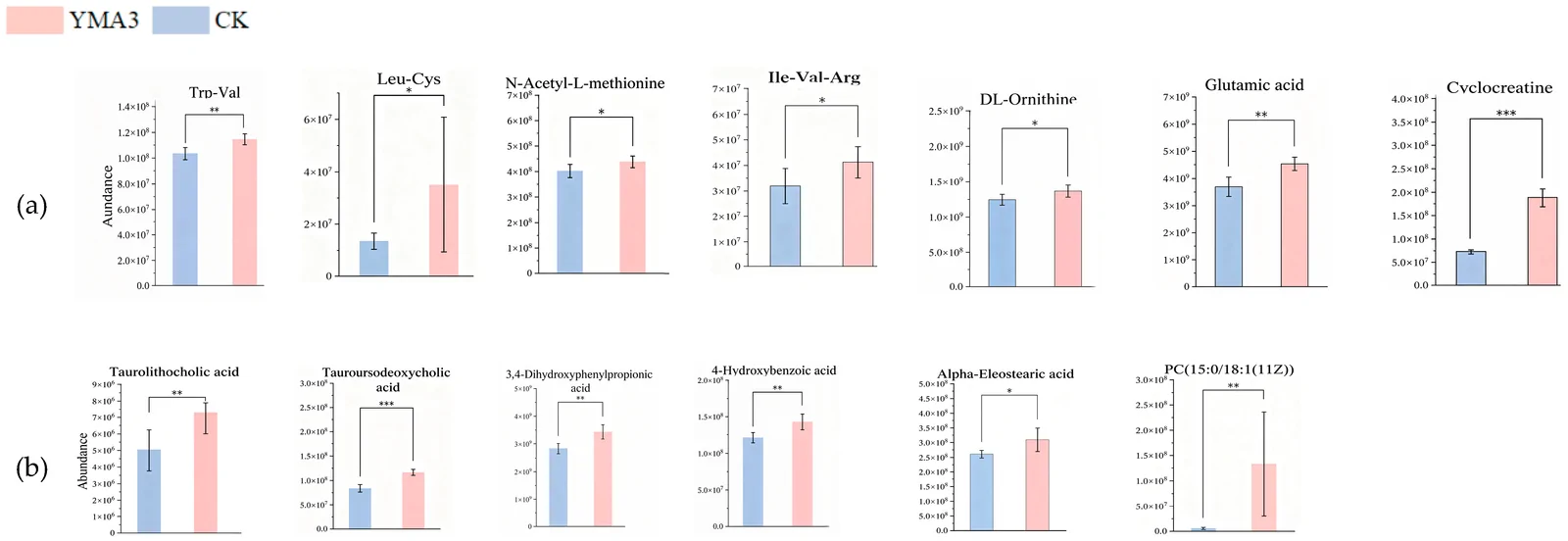
Relative abundances of selected differentially abundant metabolites with potential bioactive properties in corn stover silage. (**a**) Amino acids and dipeptides. (**b**) Antioxidant metabolites, including tauroursodeoxycholic acid (TUDCA), phosphatidylcholine (PC), and 3,4-dihydroxyphenylpropionic acid (3,4-DHPPA). Bars and error bars represent means and standard deviations of six biological replicates. *, **, and *** indicate significant differences at *p* < 0.05, *p* < 0.01, and *p* < 0.001, respectively, CK = Control.

To investigate the effects of *E. faecalis* YMA3 inoculation on metabolites after ensiling, volcano plot analysis was conducted to identify differentially abundant metabolites. A total of 2193 metabolites were detected, among which 309 exhibited significant differences (VIP > 1, *p* < 0.05), including 96 significantly increased metabolites and 213 decreased metabolites (Figure 7a,b). Increased metabolites were primarily categorized as lipids and lipid-like molecules, organic acids and their derivatives, and benzenoid compounds (Figure 8a,b). Notably, several metabolites with reported antioxidant properties in other biological systems were among the increased compounds, including tauroursodeoxycholic acid (TCDCA) and 3,4-dihydroxyphenylpropionic acid. However, we emphasize that the identification of these compounds is based on spectral matching to database entries, and their biological activity in silage matrices has not been directly demonstrated in this study (Figure 9).

**Figure 10:**
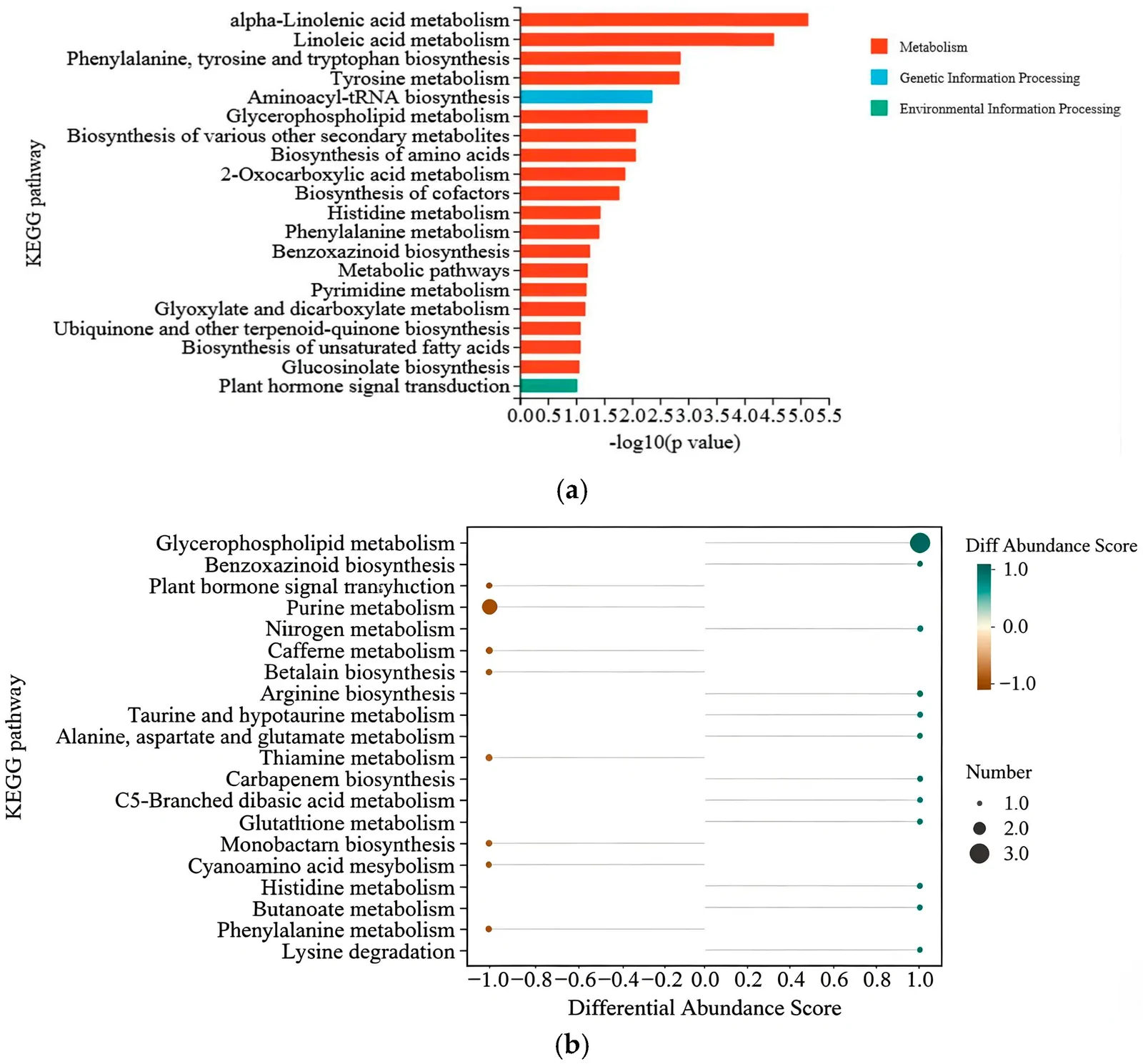
KEGG pathway enrichment analysis of differentially abundant metabolites between the *E. faecalis* YMA3-inoculated and control groups of corn stover silage. (**a**) The top 20 enriched pathways ranked by *p*-value. (**b**) Bubble plot showing pathway enrichment significance; bubble size represents the number of enriched metabolites, and color intensity indicates the significance level (−log_10_ *p*-value).

KEGG enrichment analysis was performed based on the screened differentially abundant metabolites, and the top 20 pathways ranked by ascending *p*-value were selected for visualization (Figure 10a,b). These differentially abundant metabolites were significantly enriched in pathways including alpha-linolenic acid metabolism, linoleic acid metabolism, phenylalanine, tyrosine, and tryptophan biosynthesis, tyrosine metabolism, aminoacyl-tRNA biosynthesis, glycerophospholipid metabolism, biosynthesis of various other secondary metabolites, biosynthesis of amino acids, biosynthesis of unsaturated fatty acids, and ubiquinone and other terpenoid-quinone biosynthesis. A total of 53 pathways were enriched between the Control and YMA3 groups; among these, 12 pathways were significantly enriched (*p* < 0.05), and 8 pathways were highly significantly enriched (*p* < 0.01). These 8 highly significant pathways included linoleic acid metabolism, alpha-linolenic acid metabolism, biosynthesis of phenylalanine, tyrosine, and tryptophan, tyrosine metabolism, aminoacyl-tRNA biosynthesis, glycerophospholipid metabolism, biosynthesis of various other secondary metabolites, and biosynthesis of amino acids (Table 5).

### 3.8. Correlation Between Metabolite Profiles and Fermentation Parameters

To explore potential associations between metabolic changes and silage preservation, Pearson correlation coefficients were calculated between the relative abundances of significantly differential metabolites and key fermentation parameters.

**Figure 11:**
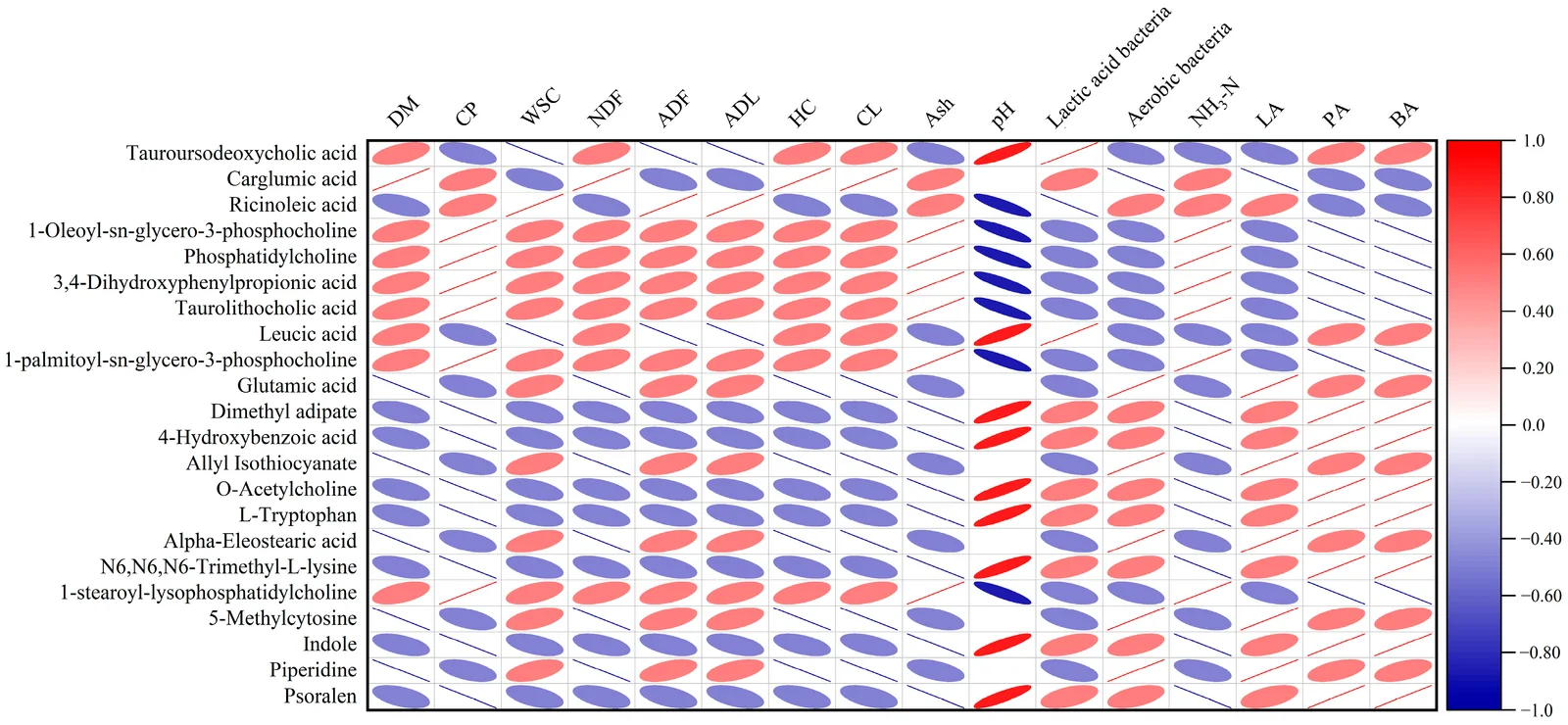
Correlation Ellipse Heatmap: Metabolites vs. Silage Fermentation Indices. An ellipse heatmap showing the correlation between metabolites (vertical axis) and silage fermentation indices (horizontal axis). Red represents positive correlation, blue represents negative correlation, and deeper color indicates stronger correlation magnitude.

As a key indicator reflecting excessive protein degradation and quality deterioration in silage, NH_3_-N showed markedly differentiated correlations with various metabolites. Some metabolites were significantly positively correlated with NH_3_-N, indicating that the accumulation of these substances coincided with excessive protein hydrolysis. Conversely, tauroursodeoxycholic acid and 3,4-dihydroxyphenylpropionic acid exhibited significantly negative correlations with NH_3_-N (*p* < 0.01) (Figure 11).

## 4. Discussion

Silage fermentation is a complex microbial process. This study elucidated the effects of *E. faecalis* YMA3 inoculation on the fermentation characteristics and metabolome of corn stover silage, providing a theoretical basis for screening target strains to regulate anaerobic fermentation of crop residues and produce high-quality silage for ruminant feeding.

### 4.1. Effects of Inoculating with E. faecalis YMA3 on the Nutritional Quality of Corn Stover Silage

Dry matter (DM) is a key indicator for evaluating silage quality, and DM loss during ensiling reflects nutrient conservation efficiency [18]. Studies have shown that DM loss associated with homolactic fermentation is typically low, ranging from 2% to 6% [19]. In the present study, no significant differences in DM content were observed among treatments at any time point (*p* > 0.05). The YMA3 group maintained stable DM content throughout ensiling, whereas the Control and F groups showed significant DM losses between days 7 and 15. This indicates that *E. faecalis* YMA3 inoculation reduces DM loss and effectively preserves nutrients during ensiling. Previous studies demonstrated that microbial inoculants can effectively preserve crude protein content [20,21]. During the initial aerobic phase, epiphytic microorganisms such as Pseudomonas spp., Escherichia coli, and Bacillus spp. utilize plant proteins and carbohydrates for growth, thereby compromising silage quality [22]. Crude protein content in the Control and F groups decreased significantly with prolonged ensiling time, whereas the YMA3 group maintained stable crude protein levels. On day 45, crude protein content in the Control group was significantly lower than in the inoculated groups, suggesting that *E. faecalis* YMA3 inoculation effectively mitigated protein loss. The higher crude protein content in the inoculated groups might also be attributed to the proliferation of inoculated bacteria, which synthesized microbial protein, thereby contributing to the total crude protein content [23], though direct measurement of bacterial protein synthesis would be required to confirm this mechanism. Improved protein preservation is particularly important for ruminant nutrition, as crude protein is often the most limiting nutrient in roughage-based diets. The improved final crude protein concentration is nutritionally significant for ruminants. As crude protein is often the most limiting nutrient in roughage-based diets, it directly affects microbial protein synthesis in the rumen and subsequent animal performance. While the magnitude of improvement was modest, even small gains in crude protein retention can translate into meaningful improvements in microbial protein supply when corn stover constitutes a major dietary component in ruminant feeding systems.

Water-soluble carbohydrates (WSC) are the primary fermentation substrates during ensiling [24]. Lactic acid bacteria utilize WSC to produce lactic acid, resulting in a decreased pH within the silage mass. With prolonged ensiling time, WSC content decreased significantly, and the WSC content in the control group was significantly higher than that in the inoculated treatments at all four time points. This pattern was attributed to efficient conversion of WSC into metabolic end products by inoculated microorganisms during ensiling [25]. Relatively high moisture content further promoted the early accumulation of acetic and butyric acids in the control group [26]. It is speculated that high water content dilutes WSC and slows down lactic acid production, delaying the rapid drop in pH, which creates a favorable environment for undesirable bacteria like acetic acid bacteria and Clostridium. These microbes metabolize the leftover substrates in the early stages of silage to produce acetic and butyric acids, which is a typical sign of poor natural fermentation when no inoculant is used.

No significant inter-group differences were detected in NDF, ADF, hemicellulose, and lignin contents throughout ensiling, which was consistent with several previous studies applying a single lactic acid bacteria inoculant on straw silage. The 45-day anaerobic fermentation period is insufficient to realize substantial degradation of lignocellulose. Lactic acid bacteria lack complete cellulase and lignase secretion systems; they only utilize small molecular water-soluble carbohydrates instead of decomposing macromolecular fiber components. Therefore, the improvement effect of a single bacterial inoculant mainly focuses on anaerobic fermentation preservation rather than destroying the dense straw stem wall structure and degrading fiber. To achieve obvious lignocellulose degradation, compound additives combining cellulase and lactic acid bacteria or longer fermentation time are required, which was not set as the research target of the current experiment.

Notably, the phenotypic advantages of YMA3 over commercial strain F were moderate in routine fermentation indicators, with non-significant differences in terminal pH and lactic acid production after 45 days of ensiling. Similar fermentation performance is reasonable because both strains belong to *E. faecalis* with homologous acid-producing pathways. However, subtle but statistically significant declines in NH_3_-N and aerobic bacterial populations reflect the unique metabolic regulation capacity of rice-derived epiphytic YMA3. Unlike commercial strains screened from a single culture medium, YMA3 was long-term adapted to the plant surface microenvironment, which enabled it to remodel the silage metabolome even under similar acidification levels. The untargeted metabolomic data further supported this viewpoint: distinct metabolic profiles were formed in YMA3 silage compared with the control groups, which indicated that the application potential of YMA3 lies in regulating secondary metabolites and antioxidative substances rather than simply accelerating lactic acid accumulation. From a practical production perspective, even small reductions in NH_3_-Ncan effectively slow continuous protein loss during long-term storage, and restraining aerobic bacteria populations greatly reduces the risk of silage spoilage after feed-out, which improves the feeding safety of ruminants.

### 4.2. Effect of Inoculating E. faecalis YMA3 on the Fermentation Quality of Corn Stover Silage

Ensiling involves LAB-mediated production of lactic acid under anaerobic conditions, resulting in a pH decline. pH serves as a critical indicator for evaluating fermentation efficiency [26,27]; lower terminal pH values indicate superior fermentation performance [28]. In the present study, pH in inoculated groups was significantly lower than in the control group at 45 days, consistent with findings by Shu [29], who reported that epiphytic *L. plantarum* strains achieved terminal pH values of 3.85–4.15 in corn silage compared to 5.32 in controls. NH_3_-Ncontent reflects protein degradation during ensiling, with elevated levels indicating extensive protein decomposition by spoilage microorganisms [30,31]. Our results demonstrated that *E. faecalis* YMA3 inoculation significantly decreased NH_3_-N content and inhibited crude protein degradation, consistent with previous studies [29,32]. At 45 days, the YMA3 group exhibited higher LAB populations and lower aerobic bacterial counts compared to other groups and the aerobic stability results indicate that YMA3 resulted in a slower increase in pH during aerobic exposure, further demonstrating the beneficial effects of *E. faecalis* YMA3 on silage quality. However, since only pH was tracked during aerobic exposure and full statistical modelling of temporal pH curves was not performed, this inference remains preliminary.

However, we caution against overinterpreting these microbial count differences as direct evidence of competitive dominance. The absence of significant changes in overall bacterial community structure suggests that YMA3’s effects may result from metabolic activity within a relatively stable community context, rather than through displacement of indigenous microbiota. This distinction is important for understanding the mode of action of inoculants.

The transient elevation of butyric acid in the Control group on day 1 suggests initial clostridial activity before the establishment of acidic conditions, though overall organic acid profiles were similar between inoculated groups at later time points.

### 4.3. Effect of Inoculating E. faecalis YMA3 on the Metabolomics of Corn Stover Silage

Untargeted metabolomics revealed differential metabolite profiles between YMA3-inoculated and control silage. Several metabolic pathways showed statistical enrichment in YMA3-treated samples, including glycerophospholipid metabolism and amino acid biosynthesis pathways. However, we emphasize that these observations are descriptive and require targeted validation to establish biological relevance.

We observed increased relative abundance of several metabolites in YMA3-treated silage, including lipids and organic acids, and amino acid derivatives. Previous mammalian cell studies reported antioxidant properties of these compounds [33,34], but such bioactivities cannot be directly extrapolated to the complex anaerobic silage matrix. The accumulation of these metabolites may arise from three potential routes: de novo synthesis by YMA3, metabolic remodeling of the indigenous microbiota induced by pH reduction, or attenuated degradation of plant intrinsic substances. Isotope labeling and targeted transcriptome analysis are needed to distinguish these possibilities, which were not conducted in this trial.

Glycerophospholipid metabolism was statistically enriched among differential metabolites. Glycerophospholipids serve as core components of microbial cell membranes [35,36], and their altered abundance may reflect the physiological adaptation of the microbiota to the acidic fermentation environment. Nevertheless, direct indicators such as membrane fluidity and integrity were not measured, so this explanation remains speculative.

Multiple amino acid-related pathways were enriched, including phenylalanine, tyrosine, and tryptophan biosynthesis. While these pathways could theoretically contribute to nitrogen metabolism and protein preservation, the direct causal link between pathway enrichment and the observed improvement in final crude protein concentration has not been established. Correlation does not imply causation, and the metabolic complexity of silage fermentation precludes definitive mechanistic conclusions from untargeted metabolomic data alone.

We detected increased relative abundance of tauroursodeoxycholic acid (TUDCA) and 3,4-DHPPA in YMA3-treated silage. When interpreting metabolomic data, a strict distinction should be made between correlation and causation. Untargeted metabolomics only identifies associations between variables. The significant correlations observed between TUDCA, 3,4-DHPPA and silage-related phenotypes merely provide correlative evidence and cannot determine causal directions. Though TUDCA and 3,4-DHPPA were reported to relieve oxidative stress in other models [37,38], three critical limitations exist in the present study: first, metabolite identification only depends on spectral matching without standard substance confirmation; second, absolute concentrations of these two compounds in silage remain unknown; third, their antioxidant and anti-aerobic-deterioration effects in the silage system have not been verified by in vitro tests. Relying solely on untargeted metabolomics cannot confirm the exact functional mechanisms of TUDCA and 3,4-DHPPA. This approach serves as a hypothesis-generating tool rather than a platform for mechanistic verification. Follow-up validations including exogenous intervention and functional assays are required to clarify the biological functions, molecular targets and regulatory pathways of these two metabolites. Thus, they are only candidate functional markers for subsequent research rather than confirmed regulatory substances.

### 4.4. Mismatch Between Community Composition and Metabolic Output in Silage Fermentation

In the silage fermentation system, microbial community composition is usually tightly coupled with the metabolite profile, and fluctuations in community structure often directly drive the remodeling of metabolic phenotypes [39]. Notably, inoculation with YMA3 significantly altered the metabolic characteristics of corn stover silage (309 differential metabolites, distinct PCA separation), but exerted no remarkable effect on the bacterial community structure.

Combined with previous data, we proposed two core explanations for this effect: first, YMA3 acted as an independent metabolic functional strain without competing with native microbes; second, rapid acidification induced by YMA3 selectively regulated the catalytic efficiency of intracellular enzymes in stable microbiota rather than reshaping community composition. This observation may imply that metabolic interactions, instead of niche competition, could dominate the processes governing silage fermentation.

Previous studies have demonstrated that *E. faecalis* YMA3 isolated from rice exerts a significant regulatory effect on the microbial community of rice silage (Shannon index reduced by 18.3%, *p* < 0.05; Bray–Curtis dissimilarity between control and YMA3 = 0.42, *p* < 0.01), whereas no distinct community shift occurred in corn stover silage (Shannon index change: 2.1%, *p* > 0.05; Bray–Curtis dissimilarity: 0.08, *p* = 0.489). Such discrepancy indicates that fermentation performance of epiphytic LAB is strongly substrate-dependent. Distinctions in fiber composition and indigenous microbiota between rice straw and corn stover create divergent physicochemical microenvironments for microbial colonization. Corn stover contains higher crude protein and lower lignin, while rice straw has greater ash content and stronger lignocellulosic recalcitrance [40]. Furthermore, the two substrates carry contrasting initial microbiomes: corn stover is enriched in taxa conducive to rapid lactic acid fermentation, whereas rice straw possesses a more diverse native community with abundant spoilage microorganisms. These traits change the niche suitability for YMA3, modulating its colonization efficiency and metabolic activity and ultimately causing inconsistent silage fermentation outcomes. In addition, the microbial interaction network in corn stover silage may be more complex and stable, resisting disturbance by exogenous inoculants [41]. Collectively, these findings emphasize that inoculant efficacy requires substrate-specific assessment.

We observed substantial metabolic remodeling without detectable shifts in overall bacterial community composition, revealing a clear decoupling between community structure and metabolic phenotype. This pattern might imply that *E. faecalis* YMA3 improves silage preservation primarily via metabolic modulation instead of niche competition. Unlike many LAB inoculants that outcompete indigenous microbes and restructure the microbiome, this rice epiphytic strain coexists with resident bacteria under anaerobic silage conditions. Rather than displacing native taxa, YMA3 rewires the fermentative metabolic network: it drives differential accumulation of lipid and amino acid metabolites and enriches top pathways including glycerophospholipid, fatty acid and amino acid metabolism. Secreted bioactive metabolites may modulate proteolysis and suppress aerobic spoilage without altering species membership of the bacterial consortium. Nevertheless, this mechanistic inference remains correlative; metatranscriptomics or stable isotope tracing will be required to verify whether YMA3 directly synthesizes these candidate metabolites or indirectly reprograms the metabolism of the indigenous microbiota.

### 4.5. Implications for Ruminant Feeding and Future Directions

While this study focused on in vitro fermentation characteristics, the ultimate goal of silage preservation is to enhance ruminant nutrition and production. The observed improvements in final crude protein concentration and aerobic stability. Suggest that YMA3-inoculated corn stover silage may offer nutritional advantages as a roughage source for ruminants. However, several limitations must be acknowledged. First, untargeted metabolomics provides relative abundance data without absolute quantification; the concentrations and bioavailability of identified metabolites in the rumen environment remain unknown. Second, the metabolic signatures observed require validation through targeted analytical methods and in vitro rumen fermentation studies to assess their impact on digestibility and fermentation kinetics. Third, the practical advantages of YMA3 over commercial inoculants, while statistically significant, were modest and require cost–benefit evaluation under commercial farm conditions. Finally, animal performance trials are needed to confirm whether the observed improvements in silage quality translate into enhanced growth performance, milk production, or feed efficiency in ruminants.

Future research should prioritize (1) targeted quantification of key metabolites using authenticated standards and MRM-based methods to verify the accumulation levels of TUDCA and 3,4-dihydroxyphenylpropionic acid in silage; (2) in vitro functional tests to validate the activities of core differential metabolites in silage matrices; (3) multi-omics integration and isotope-labeling assays to clarify the regulatory mechanism of YMA3 on silage metabolic profiling and microbial metabolic activity; (4) in vitro rumen fermentation simulation and animal feeding trials to evaluate the effects of YMA3-treated silage on ruminant nutrient digestibility and production performance; and (5) cost–benefit analysis to assess the practical application value of YMA3 as a novel silage inoculant in commercial breeding production.

One key limitation of this work is that metabolite annotation was achieved only via mass spectrum matching with public databases, lacking validation using authentic reference standards. Such tentative annotations have inherent uncertainty because structurally similar metabolites or isomers may share comparable fragment spectra. Subsequent studies need to adopt standard substances to confirm the identities of critical metabolites.

## 5. Conclusions

This study demonstrates that inoculation with *E. faecalis* YMA3, a rice epiphytic strain, effectively improves the preservation quality of corn stover silage. Specifically, YMA3 enhances final crude protein concentration and extends aerobic stability, outperforming the commercial strain F in reducing NH_3_-N concentration and aerobic bacterial counts, despite comparable lactic acid production and terminal pH between the two strains. Notably, YMA3 drives significant metabolic remodeling—evidenced by 309 differential metabolites and enriched glycerophospholipid, amino acid, and fatty acid metabolism pathways—without altering the overall bacterial community structure. This indicates YMA3 functions primarily through metabolic modulation rather than competitive exclusion, though this mechanism awaits further validation via metatranscriptomics or isotope-labeling studies. In conclusion, this work provides preliminary evidence for the potential application of *E. faecalis* YMA3 as a microbial feed additive for corn stover silage preservation, advancing the development of novel LAB inoculants for ruminant roughage utilization. Further research is needed to validate the metabolic functions of key metabolites, assess rumen fermentation characteristics, and evaluate animal performance under practical feeding conditions.

## 6. Study Limitations

This study has the following key limitations that need to be addressed in subsequent research:

- Untargeted metabolomics only provides correlative evidence and cannot establish causal relationships. The associations between differential metabolites identified in this study and silage quality are merely descriptive results, lacking direct functional verification to confirm their specific mechanisms of action.
- Metabolite identification relied solely on spectral matching without confirmation using standard substances, and the absolute concentrations of key metabolites were not determined, making it difficult to accurately quantify their actual contributions in the silage system.
- This study only focused on the corn stover silage system and did not explore the effects of differences in fiber composition and indigenous microbial communities of different substrates on the efficacy of strain YMA3, so the universality of the conclusions remains to be verified.
- The mechanism of action of YMA3 was only speculated (metabolic regulation rather than competitive exclusion), lacking direct evidence from metatranscriptomics, isotope labeling, and other approaches to clarify its specific regulatory pathways.
- In vitro functional verification assays were not conducted, so the actual biological activities of target metabolites in the silage system.

## 7. Priorities for Future Research

Based on the findings and limitations of this study, future research should focus on the following five core directions to improve the application value and mechanism analysis of strain YMA3:

- Verify the functional mechanisms of key metabolites.
- Improve metabolite quantification and identification.
- Expand substrate applicability research.
- Analyze the molecular mechanism of strain action.
- Evaluate in vivo application effects.

## Figures and Tables

**Table 1:** LC-MS/MS chromatographic parameters.

Parameter	Specification
Chromatography system	Thermo Vanquish Flex UHPLC (Thermo Fisher Scientific, Waltham, MA, USA)
Analytical column	ACQUITY UPLC HSS T3 (1.8 μm, 2.1 mm × 100 mm)
Column temperature	40 °C
Autosampler temperature	8 °C
Flow rate	0.4 mL/min
Injection volume	2 μL
Mobile phase A	0.1% formic acid in water (*v*/*v*)
Mobile phase B	Acetonitrile containing 0.1% formic acid (*v*/*v*)
Mass spectrometer	Thermo Orbitrap Exploris 120 (Thermo Fisher Scientific (Bremen) GmbH, Bremen, Germany)
Ion source	HESI electrospray source
Acquisition mode	Positive and negative electrospray ionisation modes

**Table 2:** The effect of different treatments on the nutritional quality of corn stalk silage.

Index	Treatment	Silage Days			
		1	7	15	45
DM (%FM)	CK	21.64 ± 0.07 a	21.20 ± 0.09 a	20.43 ± 0.42 b	21.43 ± 0.40 a
	YMA3	21.64 ± 0.52	21.41 ± 0.25	20.58 ± 0.85	21.37 ± 0.54
	F	21.71 ± 0.12 ab	21.83 ± 0.37 a	20.92 ± 0.31 b	20.84 ± 0.56 b
Crude protein (%DM)	CK	13.41 ± 0.26 a	13.04 ± 0.04 ab	12.67 ± 0.37 b	11.62 ± 0.04 Bc
	YMA3	13.20 ± 0.42	13.14 ± 0.38	12.54 ± 0.33	12.35 ± 0.36 A
	F	13.34 ± 0.33 a	12.60 ± 0.32 b	12.30 ± 0.15 b	12.14 ± 0.15 ABb
WSC (%DM)	CK	9.18 ± 0.40 Aa	7.57 ± 0.19 Ab	6.89 ± 0.54 Ab	5.72 ± 0.23 Ac
	YMA3	7.90 ± 0.11 Ba	6.89 ± 0.14 Bb	5.86 ± 0.24 Bc	4.34 ± 0.12 Bd
	F	7.74 ± 0.08 Ba	6.65 ± 0.29 Bb	5.67 ± 0.11 Bc	3.77 ± 0.14 Cd
NDF (%DM)	CK	51.04 ± 1.17 Ba	49.23 ± 0.74 a	49.80 ± 0.98 a	45.74 ± 0.85 b
	YMA3	52.15 ± 0.67 ABa	48.97 ± 0.80 b	48.87 ± 0.13 b	46.20 ± 0.86 c
	F	53.55 ± 0.58 Aa	48.73 ± 0.23 b	48.73 ± 0.54 b	46.58 ± 0.78 c
ADF (%DM)	CK	27.13 ± 0.83 b	27.69 ± 0.38 ab	28.48 ± 0.10 a	27.74 ± 0.57 Bab
	YMA3	27.09 ± 0.56 b	27.11 ± 0.13 b	28.07 ± 0.13 a	28.47 ± 0.08 ABa
	F	28.03 ± 0.35 b	27.24 ± 0.44 c	28.29 ± 0.24 ab	28.83 ± 0.21 Aa
ADL (%DM)	CK	2.68 ± 0.67	2.09 ± 0.21	2.41 ± 0.29	2.00 ± 0.12
	YMA3	2.45 ± 0.67	2.06 ± 0.09	2.10 ± 0.07	1.86 ± 0.13
	F	3.20 ± 0.19 a	2.38 ± 0.12 b	2.51 ± 0.14 b	2.18 ± 0.22 b
HC (%DM)	CK	23.91 ± 1.78 a	21.54 ± 0.40 ab	21.32 ± 0.93 b	18.00 ± 0.38 c
	YMA3	25.07 ± 0.46 a	21.86 ± 0.86 b	20.80 ± 0.26 b	17.73 ± 0.91 c
	F	25.52 ± 0.56 a	21.48 ± 0.60 b	20.44 ± 0.76 b	17.75 ± 0.58 c
CL (%DM)	CK	24.45 ± 0.66 b	25.60 ± 0.18 a	26.08 ± 0.19 a	25.74 ± 0.48 Ba
	YMA3	24.64 ± 0.12 d	25.05 ± 0.14 c	25.96 ± 0.06 b	26.61 ± 0.06 Aa
	F	24.83 ± 0.37 c	24.87 ± 0.45 c	25.77 ± 0.34 b	26.66 ± 0.30 Aa
Ash (%DM)	CK	8.68 ± 0.25	8.36 ± 0.14	8.58 ± 0.05	8.48 ± 0.09
	YMA3	8.59 ± 0.13	8.39 ± 0.15	8.47 ± 0.20	8.42 ± 0.23
	F	8.71 ± 0.21 a	8.39 ± 0.08 ab	8.26 ± 0.14 b	8.58 ± 0.17 ab

Note: CK = Control. Different lowercase letters in the same row indicate significant differences among different silage times under the same treatment; different uppercase letters in the same column indicate significant differences among different treatments under the same silage time (*p* < 0.05), the same applies to the table below.

**Table 3:** The Impact of Different Treatments on the Fermentation Quality of Corn Stover Silage.

Index	Treatment	Silage Days			
		1	7	15	45
pH value	CK	4.80 ± 0.01 Aa	3.77 ± 0.02 c	3.78 ± 0.00 c	3.90 ± 0.01 Ab
	YMA3	4.62 ± 0.07 Ba	3.74 ± 0.00 b	3.82 ± 0.03 b	3.83 ± 0.00 Bb
	F	4.78 ± 0.02 Aa	3.78 ± 0.01 c	3.82 ± 0.01 b	3.82 ± 0.00 Cb
LAB (log10 CFU/g FM)	CK	5.96 ± 0.05 Ba	5.95 ± 0.14 ABa	5.54 ± 0.08 b	5.72 ± 0.16 Bab
	YMA3	6.56 ± 0.33 Aa	6.39 ± 0.35 Aa	4.98 ± 0.39 b	6.58 ± 0.17 Aa
	F	6.94 ± 0.06 Aa	5.79 ± 0.06 Bc	5.04 ± 0.14 d	6.43 ± 0.13 Ab
Aerobic bacteria (log10 CFU/g FM)	CK	5.87 ± 0.14 a	5.56 ± 0.12 Bb	5.81 ± 0.03 Aa	5.90 ± 0.07 Aa
	YMA3	5.98 ± 0.28 a	5.99 ± 0.27 Aa	4.81 ± 0.24 Bb	4.87 ± 0.19 Cb
	F	6.20 ± 0.12 a	6.24 ± 0.04 Aa	5.15 ± 0.08 Bc	5.47 ± 0.07 Bb
Mold (log10 CFU/g FM)	CK	ND	ND	ND	ND
	YMA3	ND	ND	ND	ND
	F	ND	ND	ND	ND
Yeast (log10 CFU/g FM)	CK	ND	ND	ND	ND
	YMA3	ND	ND	ND	ND
	F	ND	ND	ND	ND
LA (%DM)	CK	5.18 ± 0.45 Bc	12.98 ± 2.78 b	19.78 ± 0.27 a	18.57 ± 1.40 a
	YMA3	6.14 ± 0.58 Bb	12.80 ± 2.96 a	16.06 ± 1.21 a	13.37 ± 1.74 a
	F	6.56 ± 2.47 Bb	15.59 ± 1.50 a	15.17 ± 2.80 a	17.78 ± 1.66 a
AA (%DM)	CK	2.00 ± 0.20 Aa	0.89 ± 0.21 ABc	1.46 ± 0.05 Ab	ND
	YMA3	ND	0.89 ± 0.26 AB	ND	ND
	F	0.15 ± 0.22 Bb	1.11 ± 0.05 Aa	ND	ND
PA (%DM)	CK	0.46 ± 0.05 ab	0.40 ± 0.08 b	0.56 ± 0.05 a	0.37 ± 0.05 b
	YMA3	0.38 ± 0.04 ab	0.41 ± 0.05 ab	0.46 ± 0.07 a	0.29 ± 0.07 b
	F	2.60 ± 3.05	0.45 ± 0.06	0.45 ± 0.06	0.32 ± 0.01
BA (%DM)	CK	0.51 ± 0.14 Aa	0.28 ± 0.07 b	0.34 ± 0.04 ab	0.37 ± 0.08 ab
	YMA3	0.35 ± 0.07 ABb	0.28 ± 0.05 b	0.40 ± 0.07 b	0.54 ± 0.04 a
	F	0.15 ± 0.11 B	0.32 ± 0.07	0.33 ± 0.04	0.44 ± 0.36
NH_3_-N	CK	0.53 ± 0.09 c	0.85 ± 0.06 Bb	0.76 ± 0.16 Bb	2.64 ± 0.04 Aa
	YMA3	0.56 ± 0.04 d	0.86 ± 0.08 Bc	1.04 ± 0.06 ABb	1.99 ± 0.04 Ba
	F	0.53 ± 0.06 c	1.11 ± 0.08 Ab	1.18 ± 0.14 Ab	2.47 ± 0.06 Aa

Note: “ND”means not detected.

**Table 4:** The Effect of Different Treatments and Silage Days on the Fermentation Indicators and Nutritional Indicators of corn stover.

Item	Index	Variation		
		Treatment	Ensiling Period	Interaction
Fermentation quality	pH value	**	**	**
	LA (%DM)	NA	**	NA
	AA (%DM)	**	**	**
	PA (%DM)	NA	NA	NA
	BA (%DM)	NA	NA	NA
	NH_3_-N	**	**	**
	LAB	**	**	**
	Aerobic bacteria	**	**	**
Nutritional quality	DM (%FM)	NA	**	NA
	CP (%DM)	NA	**	NA
	WSC (%DM)	**	**	*
	NDF (%DM)	NA	**	NA
	ADF (%DM)	NA	**	NA
	ADL (%DM)	*	**	NA
	HC (%DM)	NA	**	NA
	CL (%DM)	NA	**	NA
	Ash (%DM)	NA	**	NA

Note: “*” indicates significant difference (*p* < 0.05); “**” indicates highly significant difference (*p* < 0.01); “NA” indicates no significant difference.

**Table 5:** KEGG metabolic pathway annotation table.

Pathway Name	Up	Down	DEM	Total	*p*-Value
Linoleic acid metabolism	1	3	4	29	3.06 × 10^−5^
alpha-Linolenic acid metabolism	1	4	5	44	7.44 × 10^−6^
Phenylalanine, tyrosine, and tryptophan biosynthesis	2	1	3	35	0.001
Tyrosine metabolism	3	1	4	78	0.001
Aminoacyl-tRNA biosynthesis	2	1	3	52	0.004
Glycerophospholipid metabolism	3	0	3	56	0.005
Biosynthesis of various other secondary metabolites	2	1	3	66	0.009
Biosynthesis of amino acids	2	2	4	128	0.009
2-Oxocarboxylic acid metabolism	2	2	4	144	0.013
Biosynthesis of cofactors	4	2	6	330	0.017
Histidine metabolism	2	0	2	47	0.037
Phenylalanine metabolism	0	2	2	49	0.04

Note: Only the metabolic pathway of *p* < 0.05 is shown in the table.

## Data Availability

The data has been included in this article.

## References

[1] Muck R.E., Nadeau E.M.G., McAllister T.A., Contreras-Govea F.E., Santos M.C., Kung L. (2018). Silage review: Recent advances and future uses of silage additives. J. Dairy Sci..

[2] Borreani G., Bernardes T.F., Tabacco E. (2008). Aerobic deterioration influences the fermentative, microbiological and nutritional quality of maize and sorghum silages on farm in high quality milk and cheese production chains. Rev. Bras. Zootec..

[3] Okoye C.O., Wu Y., Wang Y., Gao L., Li X., Jiang J. (2023). Fermentation profile, aerobic stability, and microbial community dynamics of corn stover ensiled with *Lactobacillus buchneri* PC-C1 and *Lactobacillus plantarum* PC1-1. Microbiol. Res..

[4] Li M., Zi X., Lü R., Hu H., Tang J., Zhou H. (2020). Effects of lactic acid bacteria and cellulase on silage quality and rumen degradability of King grass Pennisetum purpureum *P. glaucum*. Chin. J. Anim. Sci..

[5] Shu G.Q., Wang H., Zhang C. (2023). The effect of tamarind-derived lactic acid bacteria on the fermentation quality of silage. Pratacultural Sci..

[6] Shu G.Q. (2023). Effects of Sanguisorba Officinalis Epiphytic Lactic Acid Bacteria on Its Silage Fermentation. Master’s Thesis.

[7] Xu D., Ding W., Ke W., Li F., Zhang P., Guo X. (2019). Modulation of metabolome and bacterial community in whole crop corn silage by inoculating homofermentative *Lactobacillus plantarum* and hetero fermentative *Lactobacillus buchneri*. Front. Microbiol..

[8] Wang Y., Sun Y., Huang K.X., Gao Y., Lin Y., Yuan B., Wang X., Xu G., Nussio L.G., Yang F. (2024). Multi-omics analysis reveals the core microbiome and biomarker for nutrition degradation in alfalfa silage fermentation. Msystems.

[9] Wu S., Huang P., Zhang C., Zhang W., Chen X., Zhang Q. (2024). Novel strategy to understand the aerobic deterioration of corn silage and the influence of *Neolamarckia cadamba* essential oil by multi-omics analysis. Chem. Eng. J..

[10] Wang X., Liu H., Wang Y., Lin Y., Ni K., Yang F. (2024). Effects of lactic acid bacteria and cellulase additives on the fermentation quality, antioxidant activity, and metabolic profile of oat silage. Bioresour. Bioprocess..

[11] Huang L., Zhang Z., Mu L., Liu X., Sun R., Gao W., Chen G., Zhou Y., Wang J., Li F. (2024). Dynamic succession of the quantity and composition of epiphytic microorganisms at different growth stages on rice surface. Front. Microbiol..

[12] Ritz K.E., Heins B.J., Moon R., Sheaffer C., Weyers S.L. (2020). Forage yield and nutritive value of cool-season and warm-season forages for grazing organic dairy cattle. Agronomy.

[13] Leveau J.H. (2019). A brief from the leaf: Latest research to inform our understanding of the phyllosphere microbiome. Curr. Opin. Microbiol..

[14] Xie Z. (2021). Effects of Urea on Dynamic Fermentation of Alfalfa Mixed Silage. Master’s Thesis.

[15] Zhang L. (2007). Feed Analysis and Feed Quality Testing Technology. Master’s Thesis.

[16] Wang Y., She X., Xie Z., Guo W., Yang M., Zhang Z. (2022). Effects of wheat bran addition ratio on the silage quality of hybrid elephant grass ‘Guimu No.1. Feed. Res..

[17] Driehuis F., Wilkinson J., Jiang Y., Ogunade I., Adesogan A. (2018). Silage review: Animal and human health risks from silage. J. Dairy Sci..

[18] Liu S. (2024). Effect of Different Bacterial Enzyme Preparations on the Quality of Protein Mulberry Silage. Master’s Thesis.

[19] Wang Y., Gao Z., Lü W., Zhang X., Fang Y., Wei P., Song M., Wang P. (2021). Effects of different microbial preparations on the quality of whole-plant silage feed from Tamarix chinensis. Feed Res..

[20] Li Y., Nishino N. (2011). Effects of inoculation of *Lactobacillus rhamnosus* and *Lactobacillus buchneri* on fermentation, aerobic stability and microbial communities in whole crop corn silage. Grassl. Sci..

[21] Lin X., Han Y., Liu W., Yao M., Huang M., Li W., Wang Y., Zhu Y., Ding D., Li Q. (2024). Meta-analysis of the effect of lactic acid bacteria additives on the nutritional and fermentation quality of maize silage. China Feed.

[22] Ávila C., Carvalho B. (2020). Silage fermentation updates focusing on the performance of micro-organisms. J. Appl. Microbiol..

[23] Amini A., Remelli A., Mojarad V.A., El Achkar M., Dell’aNno M., Rossi L., Mirra G., Di Giancamillo A., Scalvini F.G., Tedeschi G. (2025). From cheese whey to single-cell protein production: By-product valorization through acidogenic fermentation and purple phototrophic bacteria. Bioresour. Technol..

[24] Zhao J., Yin X., Li J., Wang S., Dong Z., Shao T. (2022). Effects of developmental stage and store time on the microbial community and fermentation quality of sweet sorghum silage. Ital. J. Anim. Sci..

[25] Wang S., Li J., Zhao J., Dong Z., Shao T. (2022). Dynamics of the bacterial communities and predicted functional profiles in wilted alfalfa silage. J. Appl. Microbiol..

[26] Coblentz W.K., Akins M.S., Jaramillo D.M., Cavadini J.S. (2022). Nutritive value, silage fermentation characteristics, and aerobic stability of grass-legume round-baled silages at differing moisture concentrations with and without manure fertilization and microbial inoculation. J. Anim. Sci..

[27] Ashbell G., Kipnis T., Titterton M., Hen Y., Azrieli A., Weinberg Z. (2001). Examination of a technology for silage making in plastic bags. Anim. Feed. Sci. Technol..

[28] Kung L., Shaver R. (2001). Interpretation and use of silage fermentation analysis reports. Focus Forage.

[29] Sun L., Xue Y., Xiao Y., Tian J., Zhang H., Chen S., Li X., Wang Q., Liu Y., Zhang L. (2023). Community synergy of lactic acid bacteria and cleaner fermentation of oat silage prepared with a multispecies microbial inoculant. Microbiol. Spectr..

[30] Nishino N., Hattori H. (2007). Resistance to aerobic deterioration of total mixed ration silage inoculated with and without homofermentative or heterofermentative lactic acid bacteria. J. Sci. Food Agric..

[31] Unah U.L., Inyang U.A., Jack A.A., Ekanem N.J. (2015). Silage Characteristics, Composition and Growth Performance of Wad Rams Fed Cabbage Waste Ensiled with Other Feedingstuffs. Int. J. Life Sci. Agric. Res..

[32] Moore K.J., Lechtenberg V.L., Lemenager R.P., Patterson J.A., Hendrix K.S. (1985). In vitro digestion, chemical composition, and fermentation of ammoniated grass and grass-legume silage. Agron. J..

[33] Cai Y., Ohmomo S., Ogawa M., Kumai S. (1997). Effect of NaCl-tolerant lactic acid bacteria and NaCl on the fermentation characteristics and aerobic stability of silage. J. Appl. Microbiol..

[34] Amić A., Marković Z., Klein E., Marković J.M.D., Milenković D. (2018). Theoretical study of the thermodynamics of the mechanisms underlying antiradical activity of cinnamic acid derivatives. Food Chem..

[35] Noguer M., Cerezo López A.B., Moyá Morán M.L., García-Parrilla M.C., Troncoso A.M. (2014). Synergism effect between phenolic metabolites and endogenous antioxidants in terms of antioxidant activity. Food Chem..

[36] López-Lara I.M., Geiger O. (2017). Bacterial lipid diversity. Biochim. Biophys. Acta (BBA)-Mol. Cell Biol. Lipids.

[37] Dugail I., Kayser B.D., Lhomme M. (2017). Specific roles of phosphatidylglycerols in hosts and microbes. Biochimie.

[38] Kusaczuk M. (2019). Tauroursodeoxycholate—Bile acid with chaperoning activity: Molecular and cellular effects and therapeutic perspectives. Cells.

[39] Hou Y., Luan J., Huang T., Deng T., Li X., Xiao Z., Zhan J., Luo D., Hou Y., Xu L. (2021). Tauroursodeoxycholic acid alleviates secondary injury in spinal cord injury mice by reducing oxidative stress, apoptosis, and inflammatory response. J. Neuroinflamm..

[40] Wang B., Sun H., Wang D., Liu H., Liu J. (2022). Constraints on the utilization of cereal straw in lactating dairy cows: A review from the perspective of systems biology. Anim. Nutr..

[41] Wang H. (2023). Effects of Lactic Acid Bacteria and Three Chinese Herbal Medicine Additives on the Quality, Microbial Community Structure and Metabolites of Oat Silage. Master’s Thesis.

